# A HaloTag Knock-In Resource for *In Vivo* and *In Vitro* Analysis of Endogenous Polycystin-2 Localization, Turnover, and Transport

**DOI:** 10.1681/ASN.0000001036

**Published:** 2026-02-10

**Authors:** Zhang Li, Courtney J. Haycraft, Mandy J. Croyle, Daniel Hudson, Yuan Yuan, Kristin Simanyi, Hanan Chweih Vendrame, Jun Wang, Alfiya Ibrahimbhai Kachwala, Yongjie Ma, John M. Parant, Phillip Chumley, Juling Zhou, Michal Mrug, Stephen C. Parnell, Pamela V. Tran, Hongjuan Gao, Feng Qian, Patricia Outeda, Darren P. Wallace, Terry J. Watnick, Bradley K. Yoder

**Affiliations:** 1 Department of Cell, Developmental and Integrative Biology, University of Alabama at Birmingham, Birmingham, Alabama; 2 Department of Medicine, University of Alabama at Birmingham, Birmingham, Alabama; 3 Department of Veterans Affairs Medical Center, Birmingham, Alabama; 4 Department of Biochemistry and Molecular Biology, University of Kansas Medical Center, Kansas City, Kansas; 5 Department of Internal Medicine, The Jared Grantham Kidney Institute, University of Kansas Medical Center, Kansas City, Kansas; 6 Department of Cell Biology and Physiology, University of Kansas Medical Center, Kansas City, Kansas; 7 Division of Nephrology, Department of Medicine, University of Maryland School of Medicine, Baltimore, Maryland

**Keywords:** ADPKD

## Abstract

**Key Points:**

- A HaloTag knock-in resource allows direct visualization of endogenous polycystin-2 (PC2), enabling quantitative analysis of its localization, turnover, and transport dynamics.
- PC2-HaloTag labeling establishes PC1-dependent and Tulp3-dependent control of PC2 ciliary targeting *in vivo* and in cells.

**Background:**

Polycystin-2, encoded by *PKD2*, is a cation channel essential for kidney physiology. Dysfunction of Pkd2 causes autosomal dominant polycystic kidney disease. Currently, our understanding of cystogenesis in the kidney is limited by the difficulty of visualizing the localization and molecular functions of endogenous polycystin proteins.

**Methods:**

Using clustered regularly interspaced short palindromic repeats/Cas9, we engineered a *Pkd2* HaloTag knock-in mouse (referred to as *Pkd2*^*c-Halo*^) and derived tsSV40-immortalized *Pkd2*^*c-Halo*^ renal epithelial cell lines. We optimized HaloTag labeling for *in vivo* and *in vitro* applications, demonstrating its use in confocal and live cell microscopy, pulse-chase assays, affinity isolation, and *in vivo* imaging of Pkd2 protein localization after kidney injury and in disease-relevant *Tulp3*^*R400W/R400W*^ and *Pkd1*^*null*^ mutant backgrounds.

**Results:**

Homozygous *Pkd2*^*c-Halo*^ mice were viable, fertile, and phenotypically normal, confirming that the C-terminal HaloTag did not disrupt Pkd2 function. Pkd2-c-Halo was detected by Western blotting and localized to endoplasmic reticulum and the primary cilium. Labeling occurred within 30 minutes and plateaued by 3 hours at doses of ≥2 nmol/mouse and ≥25 nM *in vivo* and in cultured cells, respectively. Pulse-chase analysis showed complete Pkd2-c-Halo turnover within 48 hours in the cilia of the choroid plexus *in vivo* and 24 hours in cultured renal epithelial cells. HaloTrap affinity resin purified the endogenous Pkd2-c-Halo from cells and tissues efficiently for protein complex analysis. While kidney injury is known to accelerate cyst formation, unilateral ureteral obstruction did not alter Pkd2-c-Halo expression or distribution. Finally, in a *Pkd1* null background or in mice homozygous for *Tulp3*^*R400W*^, an allele identified in a patient with hepatorenal cystic disease, Pkd2-c-Halo failed to localize in cilia in kidney tubule epithelium.

**Conclusions:**

The Pkd2-c-Halo mouse and derived renal epithelial cell lines enable detection of endogenous polycystin-2 *in vivo* and *in vitro*, allowing analysis of its localization, turnover under homeostatic conditions, after kidney injury, and in disease-relevant genetic backgrounds.

## Visual Abstract

**Figure:**
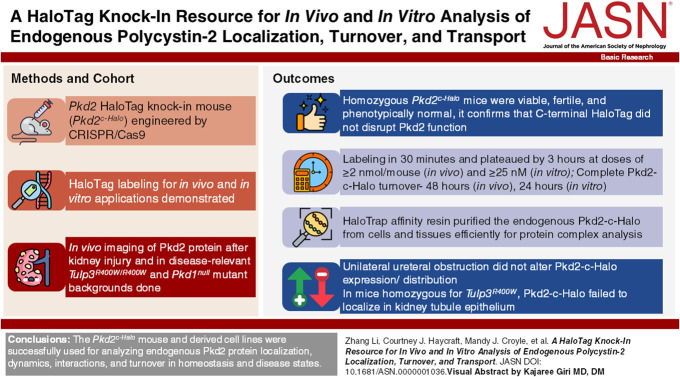

## Introduction

Autosomal dominant polycystic kidney disease is caused by mutations in *PKD1* or *PKD2* that encode for the polycystin-1 (PKD1) and polycystin-2 (PKD2) proteins, respectively.^1^ When coexpressed, PKD1 and PKD2 form a transmembrane complex consisting of one PKD1 and three PKD2 subunits that function as a nonselective cation channel.^2^ Antibody-based studies localize polycystins to various cellular sites including the cilium, endoplasmic reticulum (ER), ER–mitochondrial contact sites (mitochondrial-associated membranes [MAMs]), cell–cell junctions, exosomes, and proteolytic fragments that localize within the nucleus and mitochondria.^3–9^ Detecting endogenous PKD1 and PKD2 proteins is challenging, especially *in vivo*, because of their low expression levels that decrease with age and limited availability of robustly validated antibodies.^10–14^ Although fluorescent protein tags (*e.g*., green fluorescent protein [GFP]) have been successfully inserted into endogenous *Pkd1* and *Pkd2* loci in cells and mice, their detection requires antibody amplification preventing their use for live cell imaging.^14–19^ Consequently, much of our understanding of polycystin protein dynamics relies on exogenous overexpression systems and fixed samples.

Here, the PKD Research Consortium (PKD-RRC, https://www.pkd-rrc.org) reports the generation of new resources for detection of endogenous Pkd2 using a HaloTag inserted at the C-terminus of polycystin-2. HaloTag is a modified, approximately 33 kDa bacterial hydrolase enzyme that forms a stable covalent bond with chloroalkane group ligands under physiological conditions.^20^ The versatility of the HaloTag stems from the availability of a wide range of ligands, including membrane-permeable and nonpermeable versions.^21^ Many of the HaloTag ligands are fluorogenic with increased fluorescence on conjugation minimizing background fluorescence and reducing required washout steps.^22,23^ New ligands are being developed to incorporate biosensors for analyzing cell signaling networks in specific subcellular regions (*e.g*., cilia, MAMs), visualizing concentrations of small molecules or ions, such as Ca^2+^,^24,25^ affinity isolation, induced degradation (HaloPROTAC), proximity-based labeling, and crosslinking.^26–29^ Leveraging the mouse models and derived cell-based resources with HaloTag, we established methodologies to detect and analyze endogenous Pkd2 protein in live and fixed cells, as well as in tissues *in vivo*.

## Methods

Catalog numbers, antibody information, and all research resource identifiers (RRIDs) are included in the Supplemental Resource Table.

### Mouse Generation and Genotyping

**Figure 1:**
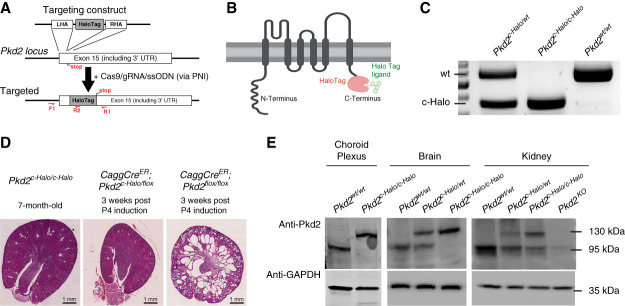
**Generation and validation of the *Pkd2***^***c-Halo***^ **mouse line.** (A) Targeting strategy to insert the HaloTag at the end of the open reading frame within exon 15 in the endogenous *Pkd2* locus. Red arrows indicate the locations of primers used for genotyping: F1 and R1 for the wild-type allele and F1 and R2 for the *Pkd2*^*c-Halo*^ allele. (B) Cartoon of the expected Pkd2-c-Halo protein structure. (C) Agarose gel showing PCR genotyping results from heterozygous and homozygous *Pkd2*^*c-Halo*^ mice and wild-type control. (D) Hematoxylin and eosin–stained section of *Pkd2*^*c-Halo/c-Halo*^ (left) kidney at age 7 months and *CAGGCre*^*ER*^; *Pkd2*^*c-Halo/flox*^ (center) 3 weeks after deletion of the conditional allele showing normal histology. Extensive cyst development is seen in *CAGGCre*^*ER*^; *Pkd2*^*flox/flox*^ mice during the same time frame. (E) Western blot showing detection of Pkd2 (110 kDa) and Pkd2-c-Halo (140 kDa) protein in tissue lysates from the choroid plexus, total brain, and kidney. Anti-GAPDH staining was used to verify similar loading between genotypes. CRISPR, clustered regularly interspaced short palindromic repeats; GAPDH, glyceraldehyde 3-phosphate dehydrogenase; gRBA, guide RNA; LHA, left homology arm; PNI, pronuclear injection; RHA, right homology arm; ssODN, single-stranded oligodeoxynucleotide; UTR, untranslated region; wt, wild-type.

To generate the *Pkd2*^*c-Halo*^ allele (*Pkd2*^*em1Bky*^; mouse genome informatics (MGI): 7461532), clustered regularly interspaced short palindromic repeats (CRISPR)/Cas9-mediated genome editing was used to insert a HaloTag coding sequence in-frame at the end of the open reading frame in exon 15 of the mouse *Pkd2* gene, immediately upstream of the termination codon. A single guide RNA (5′CACGTGTGGATTATTAGGCA) targeting exon 15 and a single-stranded repair oligo containing the HaloTag sequence flanked by 300 bp homology arms were microinjected into C57BL/6J fertilized embryos by the University of Alabama at Birmingham (UAB) Transgenic and Genetically Engineered Models Core using standard methods. A correctly targeted founder mouse was identified by PCR and mated to wild-type C57BL/6J mice for colony establishment. Correct targeting was confirmed by Sanger sequencing extending outside of the homology arms and including the full coding region. Genotyping primers (Figure 1) were F1 5′ACGTACTGCACACACTGCCCTG; R1 5′ACTGTCACCTGAAGACACGGGC; R2 5′AACATCGACGTAGTGCATGCGC.

### Mouse Husbandry and Tamoxifen Induction

Mice were housed under standard conditions with a 12-hour light/dark cycle and *ad libitum* access to food and water. All procedures were approved by the Institutional Animal Care and Use Committee (IACUC) at the University of Alabama at Birmingham (approval 09276). Genotyping was conducted using standard PCR protocols; primer sequences are also available at the PKD-RRC website. The *CAGGCre*^*ER*^ (MGI: 2182767), *Pkd2*^*flox*^ (MGI: 4843126), and *Sstr3-GFP* (MGI: 5524281) mice have been previously described.^30–32^ For conditional Pkd2 deletion and activation of the somatostatin receptor 3 fused to green fluorescent protein (Sstr3-GFP) transgene, tamoxifen was dissolved in corn oil and administered by intraperitoneal injection (6 mg/40 g body weight) on postnatal day 4 (P4). The *Tulp3*^*em1CPAM*^ (*Tulp3*^*R400W*^; MGI: 8221572) allele was generated by the UAB Center for Precision Animal Modeling (CPAM) by injection of Cas9, single guide RNA (5′GCCGTGAAAGTTCAGCACGT), and a repair template containing the R400W change directed against *Tulp3* using standard methods. The resulting mouse was backcrossed C57BL/6J mice to establish the strain, and the region of interest was sequenced to verify the desired genetic alterations.

### Western Blotting

Tissues were harvested from adult mice of the indicated genotypes. Samples were homogenized and lysed in radioimmunoprecipitation assay buffer supplemented with protease inhibitors (ThermoFisher 78430). Equal amounts (60 *µ*g) of protein were separated by sodium dodecyl-sulfate polyacrylamide gel electrophoresis on 10% Tris-Glycine gels (Bio-Rad) and transferred to polyvinylidene fluoride membranes. Membranes were blocked with Intercept Blocking Buffer (LI-COR Biotech) and probed with primary antibodies according to LI-COR–recommended protocols. Fluorescent secondary antibodies (LI-COR Biotech) were applied, and detection was performed using a LI-COR Odyssey M imaging system.

### *In Vivo* HaloTag Labeling and Live Imaging

Adult *Pkd2*^*c-Halo*^ mice were injected retro-orbitally with 1–10 nmol HaloTag ligand (see Supplemental Resource Table) in sterile PBS. Fluorophore-conjugated HaloTag ligands were obtained from Janelia Research Campus. Mice were euthanized 3 hours after ligand administration and kidneys were collected for immunofluorescence staining after fixation. Choroid plexus tissues were dissected from lateral ventricles under a stereomicroscope, mounted onto glass slides with coverslips, and imaged using a Nikon spinning disk confocal microscope.

### Immunofluorescence Staining of Fixed Tissues

Kidneys were immersion-fixed in 4% paraformaldehyde (PFA) overnight at 4°C, cryoprotected in 30% sucrose, embedded in optimal cutting temperature compound, and cryosectioned at 8 *µ*m. Sections were postfixed with 4% PFA (10′), permeabilized with 0.2% Triton X-100 (8′), and blocked for 30′ at room temperature (RT) in blocking buffer (1% BSA, 0.1% Triton X-100, 1% normal donkey serum, 0.02% sodium azide in PBS). Sections were incubated with primary antibodies diluted in blocking buffer overnight at 4°C, washed with PBS, and incubated with fluorescent secondary antibodies for 1 hour at RT. Nuclei were counterstained with Hoechst 33258 (Millipore-Sigma), and slides were mounted using IMMU-MOUNT (ThermoFisher). Confocal images were captured using Nikon Spatial Array Confocal (NSPARC) or spinning disk microscopy.

### Microscopes and Imaging

All images within a group were captured and processed under identical acquisition settings. Spinning disc confocal imaging was performed using a Nikon Ti2 Eclipse spinning disk confocal microscope (Yokogawa X1 disc, Orca Flash 4.0 Nikon camera sensor scientific complementary metal-oxide-semiconductor camera, 40× [numerical aperture (NA)=1.30] or 60× [NA=1.49] oil-immersion objective). NSPARC images were obtained on a Ti-2 Brand of Nikon confocal system microscope with a 60× (NA=1.42) oil immersion objective, and 488, 561, and 647 nm laser excitation with Nyquist sampling. For colocalization studies, confocal and NSPARC Z-stacks were acquired and deconvolved with the Richardson-Lucy algorithm with eight iterations for confocal and 15 iterations for NSPARC. Thirty images per mouse (>300 cilia per mouse) were acquired and scored manually by two independent researchers. Colocalization analysis was performed using the colocalization module in Nikon Imaging Software-Elements (v5.11) with Pearson correlation coefficient calculated from >10 regions of interest derived from at least two animals.

### Derivation and Culture of Pkd2-c-Halo Cells

Renal epithelial cells were isolated from adult kidney of *Pkd2*^*c-Halo/c-Halo*^ (RRID: CVCL_E3Y4), *CAGGCre*^*ER*^; *Pkd2*^*c-Halo/flox*^; *SSTR3-GFP* (RRID: CVCL_E3Y6), or *CAGGCre*^*ER*^; *Pkd2*^*wt/flox*^; *SSTR3-GFP* (RRID: CVCL_E3Y7) mice. Cells were transduced through lentiviral transduction using tsSV40 (Applied Biological Materials) and sorted to generate clonal lines. Cells were maintained in DMEM/F12 supplemented with PromoCell renal cell supplements and antibiotics at 33°C. After reaching confluence, cells were shifted to 37°C at least 1 day before experiments or maintained at 33°C as indicated.

### Cell Labeling and Immunofluorescence

For HaloTag labeling, cells were incubated with 50 nM HaloTag ligand diluted in DMEM/F12 plus renal cell supplements for the indicated times, followed by PBS washes. For turnover assays, cells were labeled with 50 nM Halo-JFX646 overnight, washed, and chased with 50 nM Halo-JF585 in complete media. For immunostaining, media was aspirated; cells were washed with PBS, fixed with 4% PFA (10′), permeabilized with 0.2% Triton-X100 in PBS, blocked for 30′ at RT, and stained with organelle-specific antibodies or MitoTracker Red CMXRos (ThermoFisher). After primary antibody incubation, cells were washed with PBS 3× and incubated with Alexa Fluor–conjugated secondary antibodies.

### HaloTag Pulldown Assay

Lysates from tissues or cultured cells were prepared in radioimmunoprecipitation assay buffer. HaloTag fusion proteins were isolated using the Halo-Trap Magnetic Agarose Kit according to the manufacturer's protocol. After washing, bound proteins were eluted for Western blot analysis. Parallel immunoprecipitation using anti-Pkd2 antibody was performed for comparison.

### Kidney Injury Models

Unilateral ureteral obstruction (UUO) was performed on homozygous Pkd2c-Halo mice by the UAB Small Animal Microsurgery Core (IACUC 10130): The left ureter was ligated with 5-0 silk at two sites between the bladder and kidney pelvis, cut between ligations, and the peritoneum/flank incision closed with 4-0 nylon. For unilateral ischemia-reperfusion injury (IRI; IACUC 21072), the left kidney was exposed and the left kidney pedicle clamped for 30′ followed by release for reperfusion. Mice received buprenorphine (0.05 mg/kg) and recovered on a 37°C heating pad with free access to food and water. Three days postsurgery, HaloTag ligands were administered as above, and kidneys were harvested for imaging and analysis.

### Pkd2-c-Halo Analysis in *Pkd1* and *Tulp3* Mutant Background

*Pkd2*^*c-Halo*^ mice were crossed onto the *Tulp3*^*R400W*^ and *CAGGCre*^*ER*^; *Pkd1*^*flox/flox*^ conditional mutant backgrounds. The *Pkd1*^*flox*^ allele was deleted at P4 by intraperitoneal injection tamoxifen injection (9 mg/40 g body weight). *Tulp3*^*R400W/R400W*^; *Pkd2*^*c-Halo/c-Halo*^ and *CAGGCre*^*ER*^; *Pkd1*^*flox/flox*^; *Pkd2*^*c-Halo/c-Halo*^ mice and controls received HaloTag ligand injections as above, and kidneys and choroid plexus were analyzed for Pkd2-c-Halo localization.

### Statistical Analysis

Data are presented as mean±SD. Statistical significance was determined using the unpaired Student's *t* test or one-way ANOVA followed by appropriate *post hoc* tests (Prism 9, GraphPad). *P* values are indicated in figures, and *P* < 0.05 was considered statistically significant.

## Results

### Generation and Characterization of the Pkd2 HaloTag Mouse

CRISPR/Cas9 was used to insert HaloTag into exon 15 in the endogenous *Pkd2* locus just before the translational termination sequence in single cell mouse embryos (Figure 1, A and B). The resulting founder and F1 mice were screened by PCR (Figure 1C), and precise insertion was confirmed by sequencing. Correctly targeted mice were outcrossed to C57BL/6J to establish the line *Pkd2*^*em1Bky*^ (MGI: 7461532, *Pkd2*^*c-Halo*^).

To evaluate whether insertion of the HaloTag into the C-terminus of Pkd2 disrupted function, we generated *Pkd2*^*c-Halo/c-Halo*^ homozygous mice. *Pkd2*^*c-Halo/c-Halo*^ mice were viable, fertile, and showed no overt phenotypes (Figure 1D). We further analyzed Pkd2-c-Halo function by generating *CAGGCre*^*ER*^; *Pkd2*^*c-Halo/flox*^ mice and inducing deletion of the conditional allele at postnatal day 4 (P4). In contrast to the *CAGGCre*^*ER*^; *Pkd2*^*flox/flox*^ mice that developed cystic pathology within 3 weeks, the *CAGGCre*^*ER*^; *Pkd2*^*c-Halo/flox*^ mice showed no cyst development or other kidney phenotypes (Figure 1D).

Expression of the Pkd2-c-Halo protein was confirmed by Western blot analysis of tissues isolated from *Pkd2*^*wt*^, *CAGGCre*^*ER*^; *Pkd2*^*flox/flox*^ conditional mutants, *Pkd2*^*wt/c-Halo*^, and *Pkd2*^*c-Halo/c-Halo*^ mice probed with anti-Pkd2 antibody (#3374, PKD-RRC, Figure 1E and Supplemental Figure 1). In wild-type brain and choroid plexus samples, Pkd2 was detected as an approximately 110 kDa protein. This band was largely absent from the conditional mutant, confirming specificity of the antibody. In the brain and choroid plexus samples from *Pkd2*^*c-Halo/c-Halo*^ mice, only the Pkd2-c-Halo 140 kDa protein was present, while in *Pkd2*^*wt/c-Halo*^ lysates from brain samples, both the 110 kDa Pkd2 and 140 kDa Pkd2-c-Halo proteins were detected. Similar results were observed from *Pkd2*^*wt/c-Halo*^ kidney lysates. However, in *Pkd2*^*c-Halo/c-Halo*^ kidney lysates, we frequently detected bands at approximately 110 kDa similar in size to wild-type Pkd2, which occurred with varying intensities between sample preparations in addition to the expected Pkd2-c-Halo 140 kDa band (Figure 1E and Supplemental Figure 2). We are uncertain whether these are cleavage events that result in the removal of the HaloTag or possibly consequences of protein isolation procedures. Attempts to detect Pkd2-c-Halo protein *in vivo* on Western blots using anti-Halo antibody were not successful (Supplemental Figure 2).

The use of the Pkd2-c-Halo resource was evaluated for isolation of Pkd2 protein in pulldown assays from kidney, brain, and renal epithelial cell lines derived from *Pkd2*^*c-Halo*^ mice (described below) using Halo-Trap resin. In tissues and cell lines, the Halo-Trap resin was efficient at isolating the endogenous Pkd2-c-Halo protein. Using this approach, the Pkd2-c-Halo protein was confirmed using anti-Pkd2 and anti-Halo antibodies (Supplemental Figure 3).

### *In Vivo* Detection of Pkd2-c-Halo Protein

**Figure 2:**
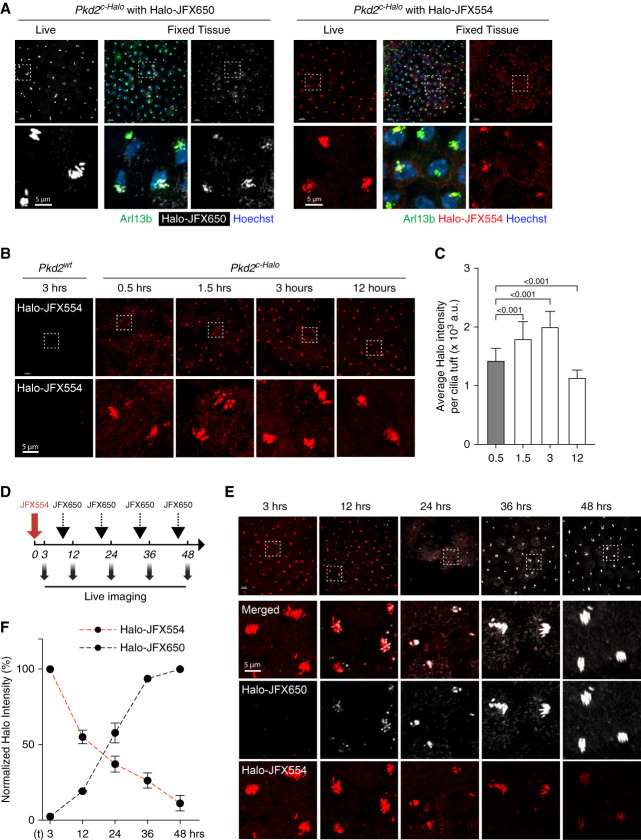
***In vivo* labeling and turnover of Pkd2-c-Halo in the choroid plexus.** (A) Confocal images of choroid plexus from *Pkd2*^*c-Halo*^ mice labeled with Halo-JFX650 (white; left) or Halo-JFX554 (red; right) 3 hours after ligand injection. Cilia are confirmed by anti-Arl13b (green) staining after fixation. (B) Confocal images of choroid plexus collected at the indicated time points after Halo-JFX554 ligand injection showing Pkd2-c-Halo signal intensity over time. (C) Quantification of fluorescence intensity from (B) over time postinjection. Significance is indicated relative to the Halo intensity at 0.5 hours (an unpaired Student's *t* test was used to compare against intensity at 0.5 hours, and statistical significance was indicated). (D) Schematic of the Pkd2-c-Halo turnover assay. Mice were injected with Halo-JFX554 (red; *t*=0) to label all Pkd2-c-Halo present at *t*=0. Three hours before isolation, mice were treated with Halo-JFX650 (white) to label all Pkd2-c-Halo produced after the initial labeling. (E) Representative confocal images from choroid plexus showing turnover of Pkd2-c-Halo (red; Halo-JFX554) and replacement with Pkd2-c-Halo (white; Halo-JFX650) over time from *Pkd2*^*c-Halo*^ mice. (F) Quantification of fluorescence signals indicating an approximately 20-hour *t*_1/2_ (defined as 50% of total Halo-ligand fluorescence) for Pkd2-c-Halo and near-complete replacement within 48 hours.

Pkd2 is known to be expressed in the choroid plexus, which includes a monolayer of ciliated epithelial cells with well-organized clusters of cilia extending into the brain ventricles, making it an ideal tissue to quickly analyze Pkd2-c-Halo localization after *in vivo* labeling.^33^ Three hours after retro-orbital injection of Halo ligand, the Pkd2-c-Halo signal was primarily evident in single to multiple small domains extending off the apical surface of the epithelium when visualized in freshly isolated choroid plexus samples as well as puncta within the cytosol at lower intensities (Figure 2A). These were confirmed to be cilia by immunofluorescence after fixation using anti-Arl13b to mark the cilium axoneme (Figure 2A). No Halo ligand signal was detected in choroid plexus from wild-type mice injected with Halo ligand (Figure 2B). Similar results were obtained with other Halo ligands tested.

To evaluate how rapidly labeling occurs *in vivo*, we collected choroid plexus at 0.5, 1.5, 3, and 12 hours post–Halo-JFX554 injection. While Pkd2-c-Halo was evident in the cilium within 0.5 hours of injection, the intensity increased until 3 hours postinjection and decreased significantly by 12 hours (Figure 2, B and C). The amount of ligand (1, 2, 5, 10 nmol/mouse) needed to obtain labeling was also titrated at 3 hours postinjection with *Pkd2*^*c-Halo*^ mice. Pkd2-c-Halo signal was readily detected in the choroid plexus cilia at 2, 5, and 10 nmol/mouse. At a dose of 1 nmol/mouse, the Halo signal was difficult to detect; therefore, we recommend 2–10 nmol/mouse for efficient and reliable detection of Pkd2-c-Halo (Supplemental Figure 4). To ensure saturation of ligand labeling, we used a dose of 10 nmol/mouse and isolated tissues 3 hours postinjection in this report.

To analyze turnover of the Pkd2-c-Halo protein in the choroid plexus cilia, Pkd2-c-Halo was initially labeled with Halo-JFX554 (*t*=0) followed by subsequent injection of Halo-JFX650 3 hours before isolation at each time point (Figure 2D). Turnover was measured as the loss of Halo-JFX554 signal and concomitant increase in Halo-JFX650 signal within cilia (Figure 2E). At 12 hours post–Halo-JFX554 injection (first ligand), Halo-JFX650 (second ligand)-labeled Pkd2-c-Halo signal became evident in the cilia with half intensity reached at approximately 20 hours and near complete replacement within 48 hours (Figure 2, E and F).

**Figure 3:**
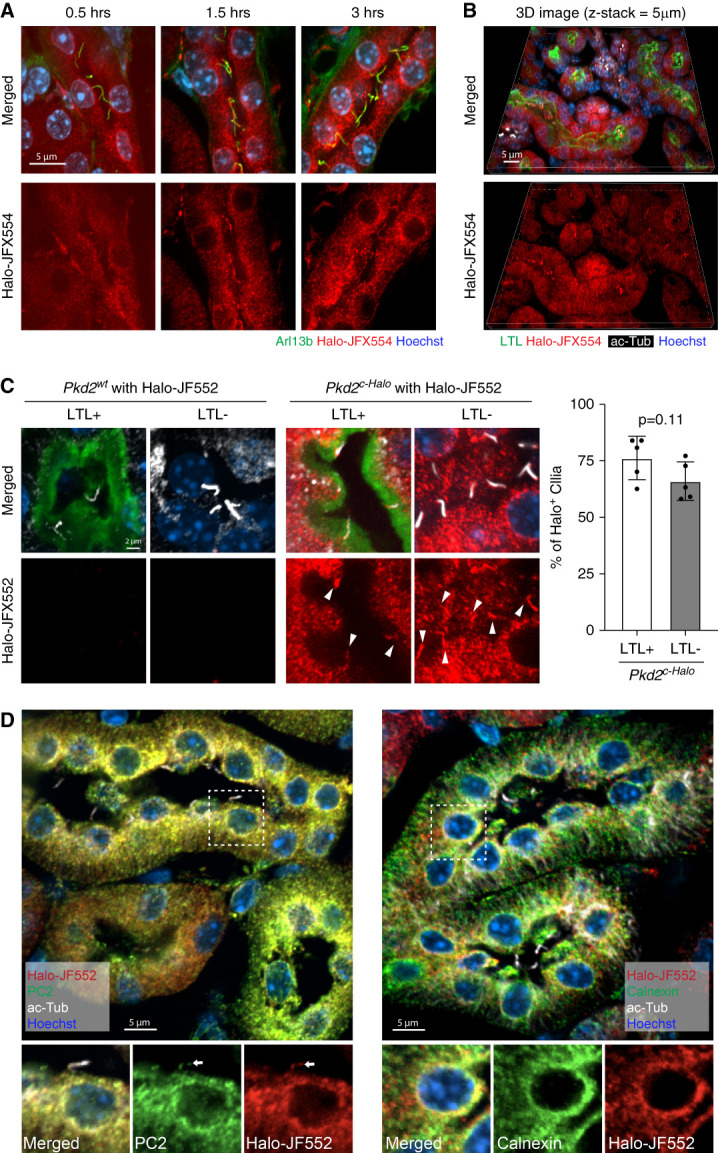
**Pkd2-c-Halo localization in kidney epithelial cells *in vivo*.** (A) Confocal images of kidney sections labeled with Halo-JFX554 (red) at the indicated time points to show labeling over time after injection. Immunofluorescence staining with anti-Arl13b (green) antibody was used to identify cilia. Images were obtained by maximum intensity projection of confocal Z-stack acquisitions, as shown in Supplemental Video 1. (B) 3D images of kidney section staining with acetylated *α*-tubulin antibody (white) and LTL (green) after injection of Halo-JF554 (red). (C) (Left) Representative confocal images of kidney section showing Pkd2-c-Halo staining (red) in both LTL+ (proximal tubule, green) and LTL− (nonproximal) tubules in *Pkd2*^*wt*^ and *Pkd2*^*c-Halo*^ kidneys. Arrow heads indicate primary cilia with Halo signaling. Cilia were labeled with acetylated *α*-tubulin. Images were obtained by maximum intensity projection of confocal Z-stack acquisitions, as shown in Supplemental Video 2 and Supplemental Video 3. (Right) Quantification of the percentage of cilia that are Halo+ in both LTL+ (proximal tubule) and LTL− (nonproximal) tubules from *Pkd2*^*c-Halo*^ mice. (D) (Left) Representative confocal images of kidney sections stained with acetylated *α*-tubulin antibody (white) and PC2 (green) after injection of Halo-JF552 (red) used for quantification of colocalization of PC2 and Pkd2-c-Halo signal, white arrow indicates cilium. Staining of *Pkd2*^*wt*^ and *Pkd2* mutant kidneys for antibody specificity are shown in Supplemental Figure 5A, including staining of wild-type Pkd2 and mutant kidneys. (Right) Representative confocal images of kidney sections stained with acetylated *α*-tubulin antibody (white) and Calnexin (green) after injection of Halo-JF552 (red) used for quantification of colocalization of ER and Pkd2-c-Halo signal. Staining of *Pkd2*^*wt*^ kidney are shown in Supplemental Figure 5B. ER, endoplasmic reticulum; LTL, lotus tetragonolobus lectin; PC2, polycystin-2.

Because most studies focus on the role of Pkd2 in the kidney and development of autosomal dominant polycystic kidney disease, we examined Pkd2-c-Halo localization in renal epithelia. As seen in the choroid plexus, Pkd2-c-Halo labeling in the kidney was detected 0.5 hours after injection and the intensity continued to increase up to 3 hours postinjection (Figure 3A). In contrast to the strong cilia enrichment observed in the choroid plexus, Pkd2-c-Halo in the kidney was seen at similar levels within the cytosol and in the cilia extending off the apical surface into the tubule lumen. This pattern is more evident in 3D images (Figure 3B) because maximum-intensity projections of Z-stacks may mask ciliary signal. Visibility of the apical cilia was increased using cardiac perfusion during fixation. To confirm the Pkd2-c-Halo signal was in the cilium, kidney sections were stained with antibodies against Arl13b (Figure 3A and Supplemental Video 1) or acetylated *α*-tubulin (Figure 3B and Supplemental Video 2). We also compared the presence of Pkd2-c-Halo in cilia from different segments of the nephron and found no difference in the percentage of Halo+ cilia in lotus tetragonolobus lectin (LTL)+ (proximal) or LTL− (all nonproximal) tubules in the kidney (Figure 3C). There was no specific signal detected following injection of the Halo ligands in wild-type mice (Figure 3C and Supplemental Video 3). To validate the Pkd2-c-Halo signal, kidneys from *Pkd2*^*c-Halo*^ mice were costained with an anti-Pkd2 antibody. Pkd2-c-Halo signal showed strong colocalization with anti-polycystin-2 staining (Pearson correlation coefficient *r*=0.83; Figure 3D, left, and Supplemental Figure 5A). In addition, Pkd2-c-Halo signal was predominantly associated with the ER, as indicated by colocalization with anti-Calnexin antibody (Pearson correlation coefficient *r*=0.65; Figure 3D, right, and Supplemental Figure 5B). The high colocalization between the Halo ligand and Pkd2 antibody indicates that the cytosolic staining, predominantly in the ER, is not Halo only but rather Halo fused with the Pkd2 protein. The high level of Pkd2-c-Halo in the cytosol made it difficult to analyze Pkd2-c-Halo turnover in the cilium of the kidney *in vivo*.

The liver is another tissue frequently affected by the loss of Pkd2 function. We analyzed liver samples after Halo ligand injection but were unable to detect Pkd2-c-Halo in cytokeratin positive bile ducts. The reason it failed in the liver is not known as the kidney and choroid plexus from the same animals matched the results included in this study.

### Detection of Pkd2-c-Halo *In Vitro*

**Figure 4:**
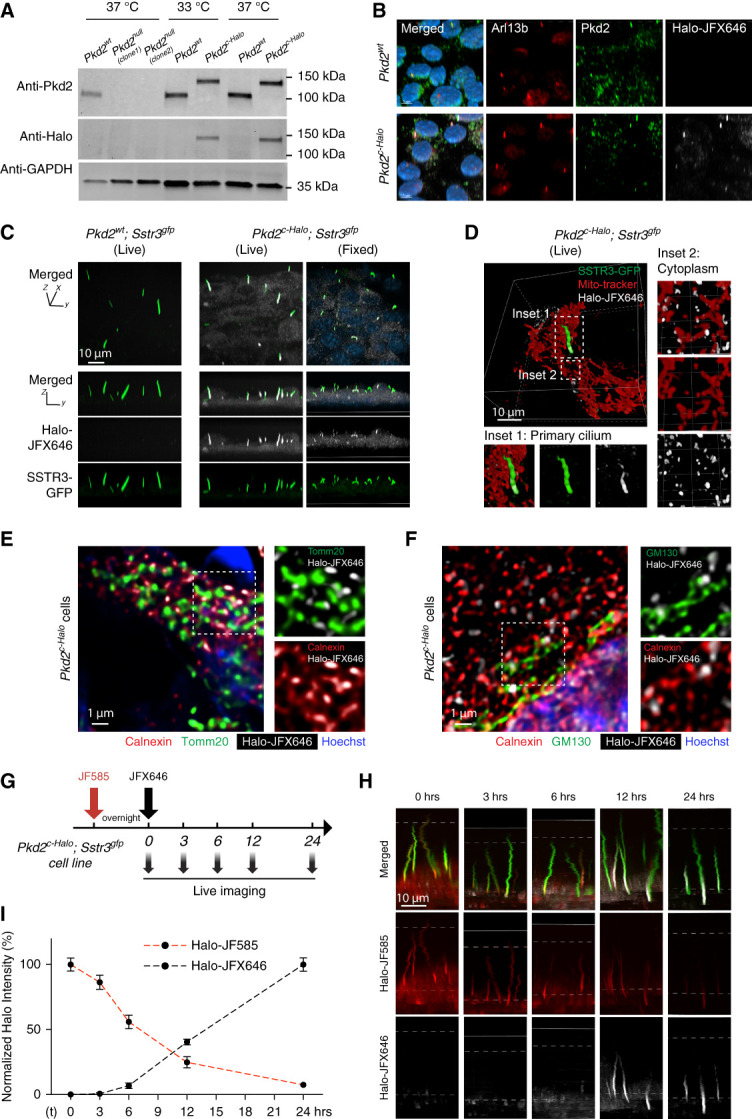
***In vitro* analysis of Pkd2-c-Halo localization and turnover in renal epithelial cells.** (A) Western blot confirming expression of Pkd2 (110 kDa) and Pkd2-c-Halo (140 kDa) proteins in *Pkd2*^*wt*^ and *Pkd2*^*c-Halo*^ cells cultured at 33°C and 37°C. The presence of the HaloTag on the Pkd2-c-Halo protein was verified by blotting with an anti-Halo antibody. *Pkd2*^*wt*^ and two *Pkd2*^*null*^ HEK293 cells from different clones were used as controls to demonstrate antibody specificity. Lysates were probed for actin as a loading control. (B) Confocal images of fixed *Pkd2*^*c-Halo*^ cells showing colocalization of Pkd2-c-Halo (white; Halo-JFX646), Arl13b (red; cilia marker), and Pkd2 (green). (C) Live cell imaging and imaging after PFA fixation showing Pkd2-c-Halo (white; Halo-JFX646) enrichment in the proximal domain of the cilium in cells coexpressing SSTR3-GFP, 3D reconstruction of the Z-stack images shown in Supplemental Video 4 and Supplemental Video 5. (D) High-resolution NSPARC confocal images of live *Pkd2*^*c-Halo*^; SSTR3-GFP cells showing proximal ciliary domain enrichment of Pkd-c-Halo (white; Halo-JFX646; inset 1) localization concentrated in the proximal cilia and cytosol (inset 2) relative to mitochondria (Mito-Tracker; red). (E) Confocal images showing colocalization of Pkd2-c-Halo (white; Halo-JFX646) with the ER marker Calnexin (green) and close proximity to mitochondria (red; Tomm20). (F) Confocal images showing minimal colocalization of Pkd2-c-Halo (white; Halo-JFX646) with the Golgi marker (red; GM130). Calnexin (red) was used to visualize the ER. (G) Schematic of the *in vitro* Pkd2-c-Halo turnover assay. Cells were incubated with Halo-JF585 (red) overnight to label all Pkd2-c-Halo protein. After washing, media containing a second Halo ligand (*t*=0; white; Halo-JFX646) was added to label newly translated Pkd2-c-Halo. (H) Representative confocal images showing reduction in Halo-JF585 and corresponding increase in Halo-JFX646 signals in cilia over time. Cells also express SSTR3-GFP (green) to visualize cilia *in vivo*. (I) Quantification of Halo-JF585 and Halo-JFX646 signal over time showing ciliary turnover of Pkd2-c-Halo an approximately 8-hour *t*_1/2_ and full signal replacement by 24 hours. GFP, green fluorescent protein; NSPARC, Nikon Spatial Array Confocal; PFA, paraformaldehyde; SSTR3-GFP, somatostatin receptor 3 fused to green fluorescent protein.

In addition to an *in vivo* resource, the PKD-RRC derived tsSV40 immortalized renal epithelial cell lines from the *Pkd2*^*c-Halo/c-Halo*^ and *Pkd2*^*c-Halo/Δ*^ mice with and without the cilia marker SSTR3-GFP (see methods) (referred to as *Pkd2*^*c-Halo*^ cells and *Pkd2*^*c-Halo*^; *Sstr3*^*gfp*^ cells, respectively). Here, we present outcomes for a line of *Pkd2*^*c-Halo*^ cells of proximal tubule origin (Supplemental Figure 6). Expression of Pkd2-c-Halo was confirmed by Western blot analysis probing with anti-Pkd2 and anti-HaloTag antibodies at the permissive (33°C) and nonpermissive temperatures (37°C). Single protein bands of the expected molecular weights were observed, and there were no obvious differences in Pkd2 or Pkd2-c-Halo expression when cells were grown at either 33°C or 37°C (Figure 4A and Supplemental Figure 7). Specificity of the antibodies were confirmed using control and CRISPR/Cas9–engineered *Pkd2*^*null*^ human embryonic kidney 293 cell line cells (Figure 4A).

Localization of Pkd2-c-Halo was analyzed in live and fixed cells after 12 hours incubation with HaloTag ligands (see Methods). There was no specific signal detected in wild-type control cells or in cells without ligand (Figure 4, B and C, and Supplemental Video 4). In *Pkd2*^*c-Halo*^ cells, Pkd2-c-Halo was located throughout the cytoplasm as seen *in vivo* but also prominently in the cilium as indicated by either colabeling with anti-Arl13b (Figure 4B) or using the included SSTR3-GFP (Figure 4C and Supplemental Video 5). In addition, we stained the *Pkd2*^*c-Halo*^ cells with anti-Pkd2 antibodies to confirm Pkd2-c-Halo localization represents Pkd2 protein *in vitro* (Figure 4B). Subcellular distribution of Pkd2-c-Halo was further analyzed by high-resolution confocal microscopy using NSPARC technology (Figure 4D). Notably, in many cilia, Pkd2-c-Halo was concentrated within the proximal region of the cilium relative to SSTR3-GFP with few cilia showing Pkd2-c-Halo signal along the length of the axoneme (Figure 4D, inset 1). The mechanism for retention of Pkd2-c-Halo in this proximal domain and its importance to Pkd2 function is not currently known. This restricted domain in the proximal region of the cilium was not readily evident in the cilia analyzed *in vivo*. After fixation, cilia were frequently shorter with the Pkd2-c-Halo domain extended along most of the axoneme (Figure 4C). The reason for the shortening after fixation is unclear.

To identify where Pkd2-c-Halo localizes in the cytoplasm *in vitro*, fixed *Pkd2*^*c-Halo*^ cells were stained with antibodies for the ER (anti-Calnexin), mitochondria (anti-Tomm20 or Mito-tracker), and Golgi (anti-GM130). Most cytoplasmic Pkd2-c-Halo puncta colocalized with Calnexin (Pearson correlation coefficient *r*=0.93, Figure 4E). Furthermore, the ER-localized domains were frequently in proximity to mitochondria, likely representing mitochondrial ER–associated membranes (MAMs) as reported previously (Figure 4, D and E, inset 2). In contrast to the ER, Pkd2-c-Halo was not frequently detected in the Golgi with GM130 staining (Pearson correlation coefficient *r*=0.13, Figure 4F).

The efficiency of labeling *in vitro* was analyzed using multiple ligands at varying concentrations. While the commercial recommendations were to use the ligands at a concentration of 250 nM, Pkd2-c-Halo was readily detected using concentrations as low as 12.5 nM of either Halo-JF585 or Halo-JFX646 (Supplemental Figure 8). Labeling was evident within 1 hour. We recommend using a ligand concentration of 50 nM in complete media overnight (>8 hours) to provide a reliable and robust Pkd2-c-Halo signal.

To evaluate turnover of Pkd2-c-Halo protein *in vitro*, *Pkd2*^*c-Halo*^; *Sstr3*^*gfp*^ cells were incubated at 37°C with Halo-JF585 for 12 hours, then washed repeatedly followed by addition of Halo-JFX646. Samples were analyzed live for changes in intensity of Pkd2-c-Halo signal in the cilium at 0, 3, 6, 12, and 24 hours after addition of the second ligand (Figure 4G). Newly translated Pkd2-c-Halo protein, as indicated by labeling with the Halo-JFX646 ligand, became evident in the cilium and cytosol between 6 and 12 hours at 37°C with near complete turnover by 24 hours. In cilia, half-fluorescence intensity for Halo-JF585 was observed at 8 hours at 37°C (Figure 4, H and I). By contrast, the turnover of Pkd2-c-Halo at 33°C was markedly slower, with half-fluorescence intensity of Halo-JF585 not occurring until 15 hours, and the presence of newly labeled Pkd2-c-Halo was not evident in the cilium until 12 hours, slower than observed at 37°C (Supplemental Figure 9).

### Analysis of Pkd2-c-Halo after Kidney Injury

**Figure 5:**
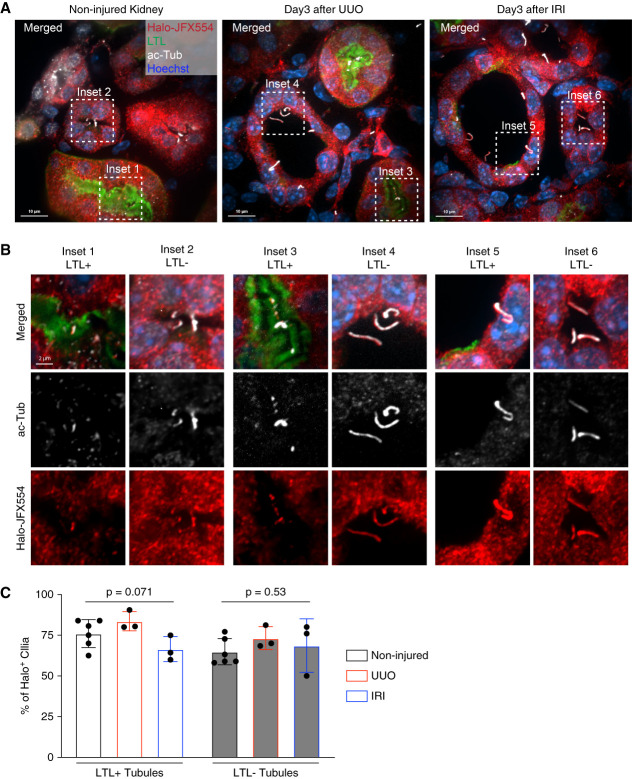
**Pkd2-c-Halo localization after kidney injury.** (A) Representative confocal images showing Pkd2-c-Halo (red; Halo-JFX554) localization in cilia (white; ac-Tub) and cytoplasm of kidney from (left) noninjured, (middle) UUO-injured, and (right) IRI-injured *Pkd2*^*c-Halo*^ mice at day 3 postinjury. Images were obtained by maximum intensity projection of confocal Z-stack acquisitions, as shown in Supplemental Video 6 and Supplemental Video 7. (B) Insets 1–6 of (A) showing Pkd2-c-Halo (red; Halo-JFX554) localization in cilia (white; ac-Tub) from LTL+ and LTL− tubules of noninjured (insets 1 and 2), UUO-injured (insets 3 and 4), and IRI-injured (insets 5 and 6) kidneys. (C) Quantification of the percentage of Halo+ cilia in LTL+ (proximal tubule) and LTL− (nonproximal) tubules of kidneys from noninjured and injured *Pkd2*^*c-Halo*^ mice at day 3 after injury. IRI, ischemia-reperfusion injury; UUO, unilateral ureteral obstruction.

Kidney injury has been shown to accelerate cyst formation in *Pkd1*, *Pkd2*, and cilia mutant mice.^34–36^ These data have led to a proposed role for cilia and the polycystins in regulating kidney injury and repair responses. To evaluate whether kidney injury alters Pkd2-c-Halo localization in renal epithelial cells, we performed two injury models in *Pkd2*^*c-Halo*^ mice, UUO, and IRI. Kidneys were collected 3 days after injury, with HaloTag ligand administered 3 hours prior to isolation. We observed that the tubules from injured kidneys were markedly dilated, and cilia were elongated as previously reported.^37^ Pkd2-c-Halo was detected in the cilium and cytosol of both injured and noninjured mice, indicating that kidney injury did not affect ciliary Pkd2-c-Halo localization (Figure 5A, Supplemental Video 6 [noninjury], Supplemental Video 7 [UUO], and Supplemental Video 8 [IRI]). In addition, in both LTL+ and LTL− tubules, the percentage of Pkd2-c-Halo positive cilia was not significantly different from injured and noninjured kidneys (Figure 5, B and C). Thus, Pkd2 trafficking and ciliary localization are not overtly affected by either UUO or IRI injury under the conditions tested, at least at the time point analyzed in this study.

### Tulp3 and Pkd1 Are Required for Pkd2-c-Halo Ciliary Localization in Renal Epithelial Cells

**Figure 6:**
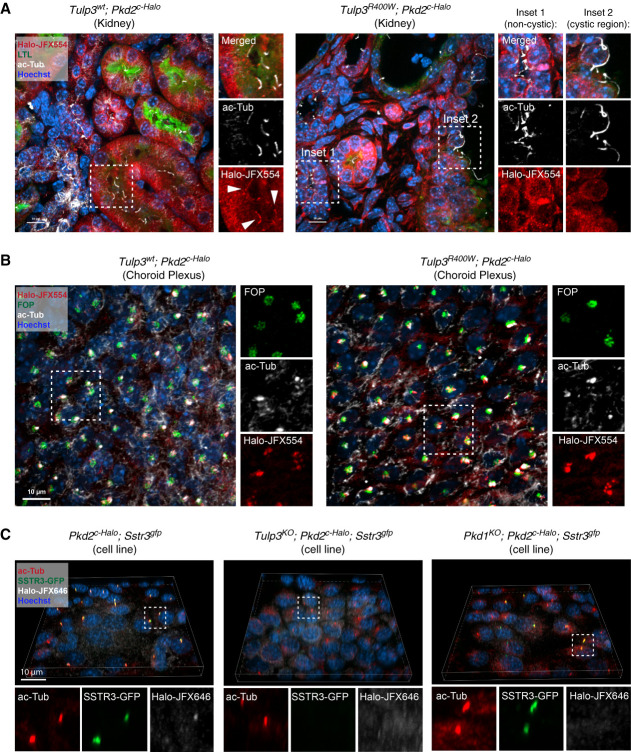
**Effect of disruption of *Pkd1* and *Tulp3* on Pkd2-c-Halo localization.** (A) Confocal images of Pkd2-c-Halo localization in kidney of control (*Pkd2*^*c-Halo*^) and *Tulp3* hypomorphic mutant (*Tulp3*^*R400W*^; *Pkd2*^*c-Halo*^) mice. Cilia were identified by staining with anti-acetylated tubulin antiserum (white). Proximal tubules were marked with LTL (green). Insets 1 and 2 represent noncystic and cystic regions of kidney tissue, respectively. Images were obtained by maximum intensity projection of confocal Z-stack acquisitions, as shown in Supplemental Video 8. (B) Confocal images of Pkd2-c-Halo localization in choroid plexus of control (*Pkd2*^*c-Halo*^) and *Tulp3* mutant (*Tulp3*^*R400W*^; *Pkd2*^*c-Halo*^) mice. Anti-acetylated tubulin staining (white) identifies the ciliary axoneme while anti-FOP (green) localizes to the base of the cilia. (C) Confocal images of Pkd2-c-Halo localization in *Pkd2*^*c-Halo*^; *Sstr3*^gfp^, *Tulp3*^*KO*^; *Pkd2*^*c-Halo*^; *Sstr3*^gfp^, and *Pkd1*^*KO*^; *Pkd2*^*c-Halo*^; *Sstr3*^gfp^ cell lines. FOP, fibroblast growth factor receptor 1 oncogene partner.

To demonstrate the utility of the *Pkd2*^*c-Halo*^ allele as a tool for studying endogenous Pkd2, we examined localization of Pkd2-c-Halo *in vivo* and in renal epithelial cell lines in the absence of either Pkd1 or Tulp3 that are known to be important for Pkd2 transport. A hypomorphic variant of *Tulp3*, *Tulp3*^*R382W*^ (*Tulp3*^*R400W*^ in mouse), was identified in a pediatric patient with a clinical diagnosis of hepatorenal fibrocystic kidney disease and submitted to the UAB CPAM for model generation to study possible disease mechanisms.^38^ Homozygous *Tulp3*^*R400W*^ mice closely mimic the index patient with cyst formation in the kidney and biliary/ductal hyperplasia in the liver (MGI: 8221628, manuscript in preparation). We also generated a *Tulp3* null mutation (*Tulp3*^*KO*^) in the *Pkd2*^*c-Halo*^; *Sstr3*^*gfp*^ cell line using CRISPR/Cas9. Disruption of Tulp3 function impaired ciliary localization of Pkd2-c-Halo *in vivo* in the kidney (Figure 6A and Supplemental Video 9) and in *Tulp3*^*KO*^ CRISPR–engineered renal epithelial cells (Figure 6C and Supplemental Figure 10), while localization in choroid plexus cilia was not perturbed (Figure 6B). SSTR3-GFP was also lost from the cilium in *Tulp3*^*KO*^ cells. This agrees with previous literature indicating that Tulp3 is required for localization of several proteins to the ciliary axoneme, including Pkd2.^39,40^

Pkd1 is also known to be essential for the ciliary localization of Pkd2. To determine whether the localization of Pkd2-c-Halo is disrupted in the absence of Pkd1, we used CRISPR/Cas9 to disrupt *Pkd1* in the *Pkd2*^*c-Halo*^; *Sstr3*^*gfp*^ cell line. As seen after disruption of Tulp3, Pkd2-c-Halo failed to localize to cilia in the absence of Pkd1, although the localization of SSTR3-GFP was not affected (Figure 6C and Supplemental Figure 10).

## Discussion

The PKD-RRC has generated and characterized a novel *Pkd2*^*c-Halo*^ mouse model and associated renal epithelial cell lines. The *Pkd2*^*c-Halo*^ mouse was generated through insertion of a HaloTag just prior to the translational stop codon in exon 15 of the endogenous *Pkd2* locus. *Pkd2*^*c-Halo/c-Halo*^ or *CaggCre*^*ER*^; *Pkd2*^*c-Halo/flox*^ conditional mice were viable, fertile, and displayed no overt phenotypes, indicating there is no effect of HaloTag insertion on Pkd2 function or expression.

The *Pkd2*^*c-Halo*^ mouse and cell lines were confirmed to be valuable tools for *in vivo* and *in vitro* studies of the endogenous Pkd2 protein. Using multiple HaloTag ligands, the Pkd2-c-Halo protein was visualized in the cytosol and cilia of the choroid plexus and kidney and in immortalized kidney cells in culture. The Pkd2-c-Halo protein within the cytosol *in vivo* and *in vitro* overlapped at sites of ER–mitochondria juxtaposition, suspected to be ER–MAMs as reported previously for Pkd2.^4^ In cultured renal epithelial cells, Pkd2-c-Halo protein was largely restricted to the proximal region of most cilia. The proximal enrichment of Pkd2-c-Halo was not evident in cilia when visualized *in vivo* in either the choroid plexus or kidney or following kidney injury, but the high levels of cytosolic Pkd2-c-Halo in the kidney and shorter average length of cilia made this difficult to evaluate. To better visualize ciliary Pkd2-c-Halo *in vivo*, we found cardiac perfusion widened luminal space and improved visibility. In addition, the cilia observed on cultured *Pkd2*^*c-Halo*^ cells in culture were longer than the reported average length of cilia in the renal epithelial cells *in vivo*. It is not currently known whether this proximal restriction is due to the extended length of cilia or whether this reflects a Pkd2 enrichment zone with functional significance. Studies to determine whether the Pkd2 enrichment zone correlates with the Inversin compartment or differences in lipid composition known to be present in the proximal versus distal regions of the cilium are underway. In addition, it will be important to define whether this proximal restriction is dynamic and changes in response to different stimuli (*e.g*., flow, injury, *etc.*).

The dynamics of Pkd2-c-Halo labeling *in vivo* and *in vitro* demonstrated that ligand uptake and labeling of the Pkd2-c-Halo protein was efficient. *In vivo*, Pkd2-c-Halo was detected by 30′ postinjection and plateaued by 90’. *In vitro*, labeling occurred within 1 hour. Our studies on ligand dosage and label duration establish conditions for reliable and efficient *in vivo* and *in vitro* detection.

We also analyzed Pkd2-c-Halo turnover in the cilium of the choroid plexus. *In vivo*, new Pkd2-c-Halo signal could be detected in cilia at approximately 12 hours with nearly complete turnover of Pkd2-c-Halo in 48 hours. Ligand titration and temporal analysis indicated that at our recommended ligand concentrations, the labeling strategy is fully saturating. While it is possible that a pool of Pkd2-c-Halo protein is sequestered and not labeled with the initial dose, there is no precedent for the presence of a pool of protein that would be unlabeled in the initial experiments that would alter the conclusions. We have not yet evaluated whether similar kinetics occurred in the kidney, in part because of the challenge of visualizing cilia in the kidney relative to the amount of Pkd2-c-Halo in the cytosol. In cell culture, newly labeled Pkd2-c-Halo protein is first seen within 12 hours with near complete turnover by 24 hours. The turnover was affected by temperature, or possibly differentiation state of the cells, with turnover occurring slower in cells cultured at 33°C.

We used the *Pkd2*^*c-Halo*^ mouse line to assess whether injury induced by UUO or IRI affects the localization of endogenous Pkd2. Our analysis indicated that injury did not affect Pkd2-c-Halo expression or its localization in the cilium or cytosol. We analyzed this 3 days postinjury, a major time point in the injury response in mice.^41^ It is possible that analysis at other time points may reveal differences in Pkd2 localization or trafficking between injured and noninjured states, and these studies are ongoing.

Finally, we demonstrated the use of the *Pkd2*^*c-Halo*^ mouse line to analyze transport defects associated with disease states. In collaboration with the UAB CPAM, we generated a mouse line carrying a *Tulp3*^*R400W*^ mutation. Tulp3 is important for trafficking multiple proteins into the cilium.^42,43^ This mouse model was generated because of a hepatorenal cystic disorder patient case submitted to CPAM for analysis (manuscript in preparation). As in the patient, homozygous *Tulp3*^*R400W*^ mice developed kidney cysts and biliary and bile duct hyperplasia. Tulp3's relationship with cyst development is complex, with other mutations having positive and negative effects on the rate and severity of cyst formation depending on context.^38,40,42,44,45^ Mutations in *Tulp3* promote cyst formation during kidney development, but attenuate cyst development in *Pkd1* or *Pkd2* mutant backgrounds in adult-induced conditional mutants.^40,45^ In mice, a previous study showed that *Tulp3*^*K407I*^ mutations, corresponding to a Joubert Syndrome patient, have a modest 30% reduction in Pkd2 protein expressed in cilia on the kidney epithelium.^45^ In the *Tulp3*^*R400W*^ model, we were unable to detect Pkd2-c-Halo in the cilium in the kidney. These data indicated that a possible cause for cyst development in the CPAM patient case is a defect in trafficking of Pkd2 into kidney cilia. This was not observed in the choroid plexus cilia, although other Tulp protein family members are expressed in choroid plexus that could mediate Pkd2 trafficking. Similarly, in the absence of Pkd1, Pkd2-c-Halo failed to localize in the cilium in a renal epithelial cell line. A role for Pkd1 in Pkd2 trafficking has been reported previously and is confirmed here.^17,46^ In the context of the Tulp3 mutation, failed Pkd2 ciliary localization as a driver of cyst development should be taken cautiously as Tulp3 is involved in trafficking of multiple proteins into the cilium, several of which cause cyst development when disrupted.^40,44^

In summary, we have generated an important new resource for the PKD research community and established methodology for *in vivo* and *in vitro* analysis. We demonstrated the utility of the *Pkd2*^*c-Halo*^ mouse model and its cell line derivatives to study endogenous Pkd2 in live and fixed cells and tissues. Collectively, these data established the *Pkd2*^*c-Halo*^ mouse and cells as new platforms for the *in vivo* and *in vitro* exploration of Pkd2 localization, trafficking, turnover, protein interactions, and how localization may change in responses to genetic mutations or disease states associated with cyst formation. Additional engineering in these *Pkd2*^*c-Halo*^ resources will facilitate the analysis of how patient variants impact these processes and will open new avenues for testing modes of action of candidate therapeutic drugs. We are making these resources readily available from the PKD-RRC.

## Supplementary Material

## Data Availability

Original data generated for the study will be made available upon reasonable request to the corresponding author. Data Type: Image Data; Research Protocols; Published Material; Observational Data; Raw Data/Source Data. Reason for Restricted Access: No large datasets are included in this project, all the experimental data are available upon reasonable request.

## Declarative Statements

All animal experiments were conducted in accordance with the NIH Guide for the Care and Use of Laboratory Animals or an equivalent standard that meets or exceeds the ethical and welfare requirements outlined in the NIH Guide. All protocols were approved by the appropriate institutional animal care and use committee.

## Supplemental Material

This article contains supplemental material online, published as provided by the authors, at http://links.lww.com/JSN/F695, http://links.lww.com/JSN/F696, http://links.lww.com/JSN/F697, http://links.lww.com/JSN/F698, http://links.lww.com/JSN/F699, http://links.lww.com/JSN/F700, http://links.lww.com/JSN/F701, http://links.lww.com/JSN/F702, http://links.lww.com/JSN/F703, http://links.lww.com/JSN/F704, http://links.lww.com/JSN/F705.

Supplemental Figure 1. Full-length immunoblots of Western blot (WB) results for Figure 1E.

Supplemental Figure 2. Additional independent full length immunoblots from separate WB experiments with antibodies against PC2 and Halo. Blots were probed for actin or glyceraldehyde 3-phosphate dehydrogenase as a loading control.

Supplemental Figure 3. Pulldown analysis with HaloTrap resin with samples from tissue and cell lines.

Supplemental Figure 4. Pkd2-c-Halo signal in choroid plexus from *Pkd2*^*c-Halo*^ mice with varying doses of Halo-JFX554.

Supplemental Figure 5. (A) Representative confocal images of kidney sections stained with acetylated *α*-tubulin antibody (white) and PC2 (green) after injection of Halo-JF552 (red) used for quantification of colocalization of PC2 and Pkd2-c-Halo signal, white arrow indicates cilium. (B) Representative confocal images of kidney sections stained with acetylated *α*-tubulin antibody (white) and Calnexin (green) after injection of Halo-JF552 (red) used for quantification of colocalization of ER and Pkd2-c-Halo signal.

Supplemental Figure 6. Proximal tubule origin of *Pkd2*^*c-Halo*^ cell line.

Supplemental Figure 7. Full-length immunoblots of WB results for Figure 4A.

Supplemental Figure 8. Pkd2-c-Halo signal in *Pkd2*^*c-Halo*^ cell line with different concentrations of Halo ligands incubations.

Supplemental Figure 9. Pkd2-c-Halo turnover in *Pkd2*^*c-Halo*^; *Sstr3*^*gfp*^ cell line at 33°C.

Supplemental Figure 10. Loss of ciliary localization of Pkd2-c-Halo in *Pkd2*^*c-Halo*^; *Sstr3*^*gfp*^ cells with Pkd1 deletion or Tulp3 mutation with live cell imaging.

Supplemental Resource Table (Excel).

Supplemental Video 1. Pkd2-Halo kidney labeled with HaloTag ligand JFX554; Arl13b marks primary cilia. Related to Figure 3.

Supplemental Video 2. Pkd2-Halo kidney labeled with HaloTag ligand JF552; LTL marks proximal tubules; acetylated *α*-tubulin marks primary cilia. Related to Figure 3.

Supplemental Video 3. Pkd2-WT kidney incubated with HaloTag ligand JF552 (negative control); LTL marks proximal tubules; acetylated *α*-tubulin marks primary cilia. Related to Figure 3.

Supplemental Video 4. Pkd2-WT; SSTR3-GFP cells incubated with HaloTag ligand JFX646. Related to Figure 4.

Supplemental Video 5. Pkd2-Halo; SSTR3-GFP cells incubated with HaloTag ligand JFX646. Related to Figure 4.

Supplemental Video 6. Noninjured Pkd2-Halo kidney labeled with HaloTag ligand JFX554; LTL marks proximal tubules; acetylated *α*-tubulin marks primary cilia. Related to Figure 5.

Supplemental Video 7. UUO-injured Pkd2-Halo kidney labeled with HaloTag ligand JFX554; LTL marks proximal tubules; acetylated *α*-tubulin marks primary cilia. Related to Figure 5.

Supplemental Video 8. IRI-injured Pkd2-Halo kidney labeled with HaloTag ligand JFX554; LTL marks proximal tubules; acetylated *α*-tubulin marks primary cilia. Related to Figure 5.

Supplemental Video 9. Tulp3 mutant Pkd2-Halo kidney labeled with HaloTag ligand JFX554; LTL marks proximal tubules; acetylated *α*-tubulin marks primary cilia. Related to Figure 6.
