# Supplementary material for: A HaloTag Knock-In Resource for In Vivo and In Vitro Analysis of Endogenous Polycystin-2 Localization, Turnover, and Transport
Source: J Am Soc Nephrol. 2026 Feb 10;37(8):1674–88. doi: 10.1681/ASN.0000001036 (PMC13381075; doi:10.1681/ASN.0000001036)
Supplement: Supplementary file 2 [file jasn-37-1674-s002.pdf]

Supplemental Material Table of Contents

Supplemental Figure 1. Full length immunoblots of WB results for Figure 1E.

Supplemental Figure 2. Additional independent full length immunoblots from separate WB experiments with antibodies against PC2 and Halo. Blots were probed for actin or GAPDH as a loading control.

Supplemental Figure 3. Pulldown analysis with HaloTrap resin with samples from tissue and cell lines.

Supplemental Figure 4. Pkd2-c-Halo signal in choroid plexus from *Pkd2*$^{c\text{-}Halo}$ mice with varying doses of Halo-JFX554.

Supplemental Figure 5. (A) Representative confocal images of kidney sections stained with acetylated α-tubulin antibody (white) and PC2 (green) following injection of Halo-JF552 (red) used for quantification of co-localization of PC2 and Pkd2-c-Halo signal, white arrow indicates cilium. (B) Representative confocal images of kidney sections stained with acetylated α-tubulin antibody (white) and Calnexin (green) following injection of Halo-JF552 (red) used for quantification of co-localization of ER and Pkd2-c-Halo signal.

Supplemental Figure 6. Proximal tubule origin of *Pkd2*$^{c\text{-}Halo}$ cell line.

Supplemental Figure 7. Full length immunoblots of WB results for Figure 4A.

Supplemental Figure 8. Pkd2-c-Halo signal in *Pkd2*$^{c\text{-}Halo}$ cell line with different concentrations of Halo ligands incubations.

Supplemental Figure 9. Pkd2-c-Halo turnover in *Pkd2*$^{c\text{-}Halo}$*;Sstr3*^{gfp} cell line at 33°C.

Supplemental Figure 10. Loss of ciliary localization of Pkd2-c-Halo in *Pkd2*$^{c\text{-}Halo}$*;Sstr3*^{gfp} cells with Pkd1 deletion or Tulp3 mutation with live cell imaging.

Supplemental Resource Table (Excel).

Supplemental Video 1. Pkd2-Halo kidney labeled with HaloTag ligand JFX554; Arl13b marks primary cilia. Related to Figure 3.

Supplemental Video 2. Pkd2-Halo kidney labeled with HaloTag ligand JF552; LTL marks proximal tubules; acetylated α-tubulin marks primary cilia. Related to Figure 3.

Supplemental Video 3. Pkd2-WT kidney incubated with HaloTag ligand JF552 (negative control); LTL marks proximal tubules; acetylated α-tubulin marks primary cilia. Related to Figure 3.

Supplemental Video 4. Pkd2-WT; SSTR3-GFP cells incubated with HaloTag ligand JFX646. Related to Figure 4.

Supplemental Video 5. Pkd2-Halo; SSTR3-GFP cells incubated with HaloTag ligand JFX646. Related to Figure 4.

Supplemental Video 6. Non-injured Pkd2-Halo kidney labeled with HaloTag ligand JFX554; LTL marks proximal tubules; acetylated α-tubulin marks primary cilia. Related to Figure 5.

Supplemental Video 7. UUO-injured Pkd2-Halo kidney labeled with HaloTag ligand JFX554; LTL marks proximal tubules; acetylated α-tubulin marks primary cilia. Related to Figure 5.

Supplemental Video 8. IRI-injured Pkd2-Halo kidney labeled with HaloTag ligand JFX554; LTL marks proximal tubules; acetylated α-tubulin marks primary cilia. Related to Figure 5.

Supplemental Video 9. Tulp3 mutant Pkd2-Halo kidney labeled with HaloTag ligand JFX554; LTL marks proximal tubules; acetylated α-tubulin marks primary cilia. Related to Figure 6.

Supplemental Figure 1. Full length immunoblots of WB results for Figure 1E

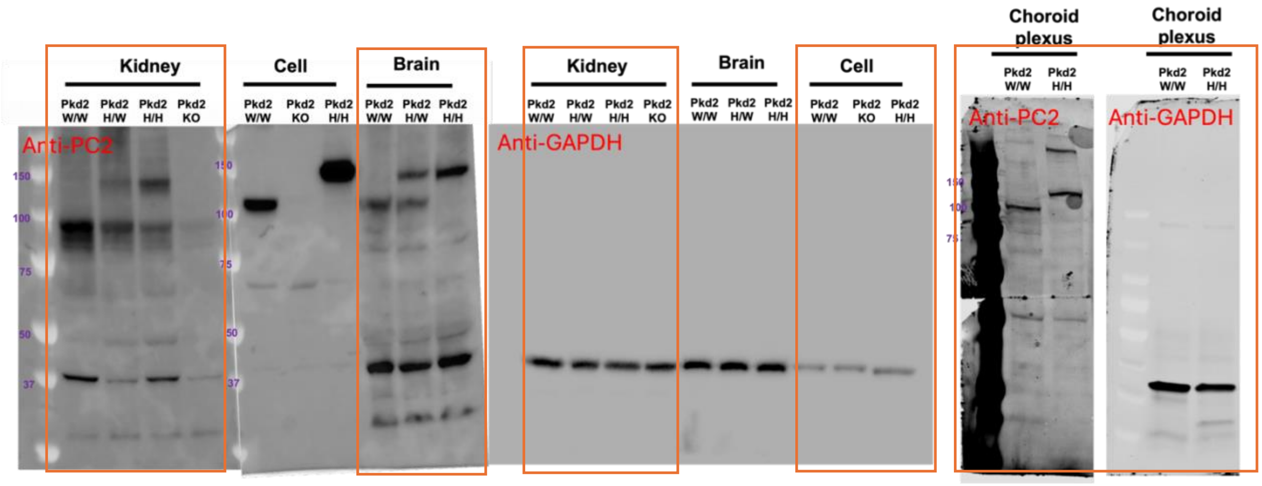

Supplemental Figure 2. Additional independent full length immunoblots from separate WB experiments with antibodies against PC2 and Halo. Blots were probed for actin or GAPDH as a loading control. **(A)** Lysis with RIPA buffer with proteinase inhibitors, 10 sec sonication, 60 µg protein (tissues) and 20 µg (cells) loaded, denatured at 37°C for 15 min with loading buffer. **(B)** Lysis with RIPA buffer with proteinase inhibitors, 10 sec sonication, 60 µg protein (kidney) and 20 µg (cells) loaded, denatured at 37°C for 15 min with loading buffer. **(C)** Lysis with RIPA buffer with proteinase inhibitors, 10 sec sonication, 60 µg protein loaded, denatured at 37°C for 15 min with loading buffer. **(D)** Lysis with 20 mM Na phosphate, 150 mM NaCl, 1 mM EDTA, 10% glycerol, 1% Triton X-100 for 60 min at 4°C, no sonication, 80 µg protein loaded, denatured at 37°C for 15 min with loading buffer.

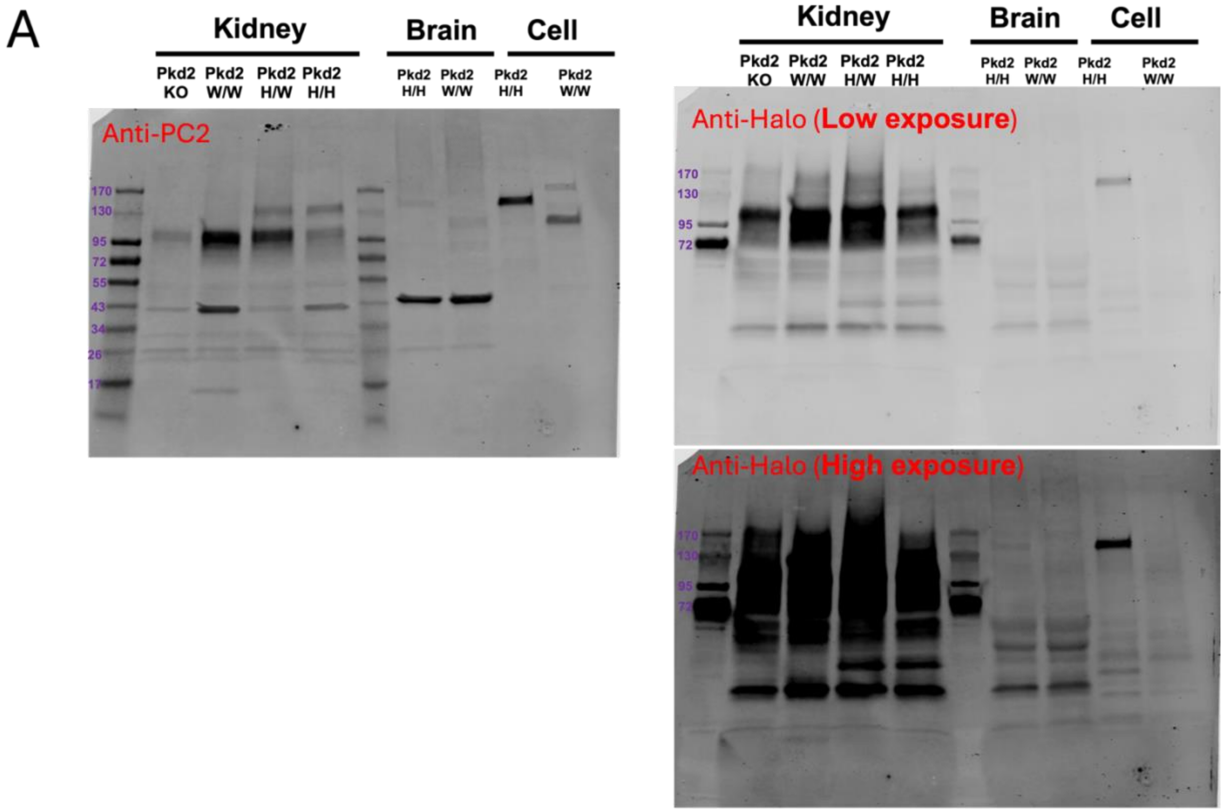

B

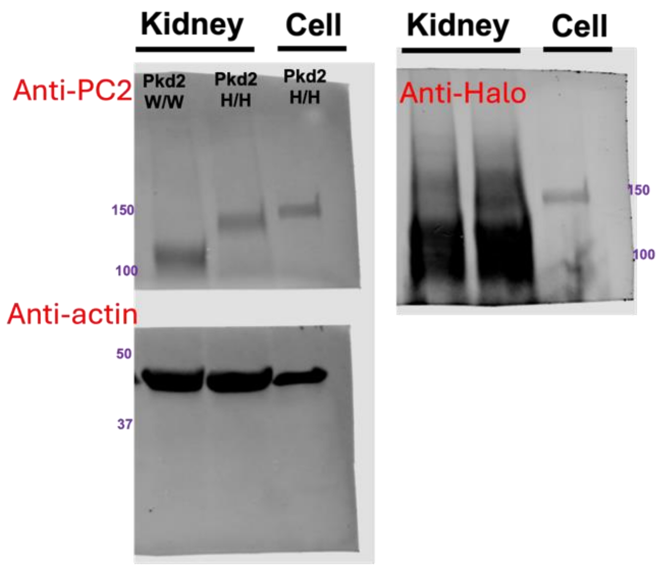
Kidney
Cell
Kidney
Cell
Anti-PC2
Pkd2 W/W
Pkd2 H/H
Pkd2 H/H
150
100
Anti-Halo
150
100
Anti-actin
50
37

C

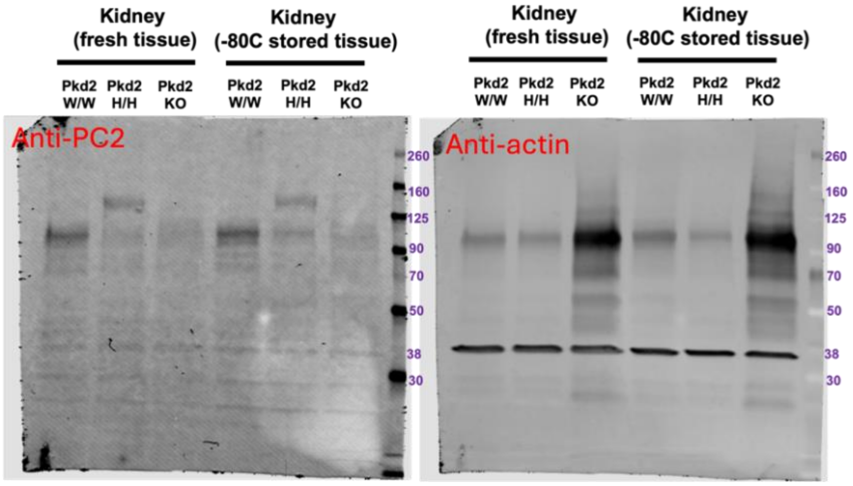
Kidney (fresh tissue)
Kidney (-80C stored tissue)
Pkd2 W/W
Pkd2 H/H
Pkd2 KO
Pkd2 W/W
Pkd2 H/H
Pkd2 KO
Anti-PC2
260
160
125
90
70
50
38
30
Kidney (fresh tissue)
Kidney (-80C stored tissue)
Pkd2 W/W
Pkd2 H/H
Pkd2 KO
Pkd2 W/W
Pkd2 H/H
Pkd2 KO
Anti-actin
260
160
125
90
70
50
38
30

D

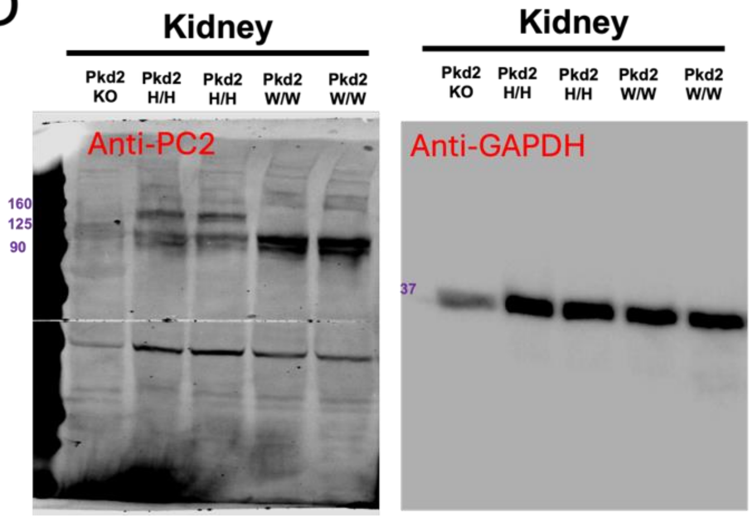
Kidney
Pkd2 KO
Pkd2 H/H
Pkd2 H/H
Pkd2 W/W
Pkd2 W/W
Anti-PC2
160
125
90
Kidney
Pkd2 KO
Pkd2 H/H
Pkd2 H/H
Pkd2 W/W
Pkd2 W/W
Anti-GAPDH
37

Supplemental Figure 3. Pulldown analysis with HaloTrap resin with samples from tissue and cell lines. Western blot of input and bound (elution) Pkd2 and Pkd2-c-Halo from tissue **(A)** of *Pkd2*^{wt} and *Pkd2*$^{c\text{-}Halo}$ brain and kidney and **(B)** *Pkd2*$^{c\text{-}Halo}$ cells. Only Pkd2-c-Halo was bound by the HaloTrap resin and is detected by both Pkd2 and Halo antibodies. While Pkd2 was detected in lysates from tissues and cells, it was not pulled down by the HaloTrap resin.

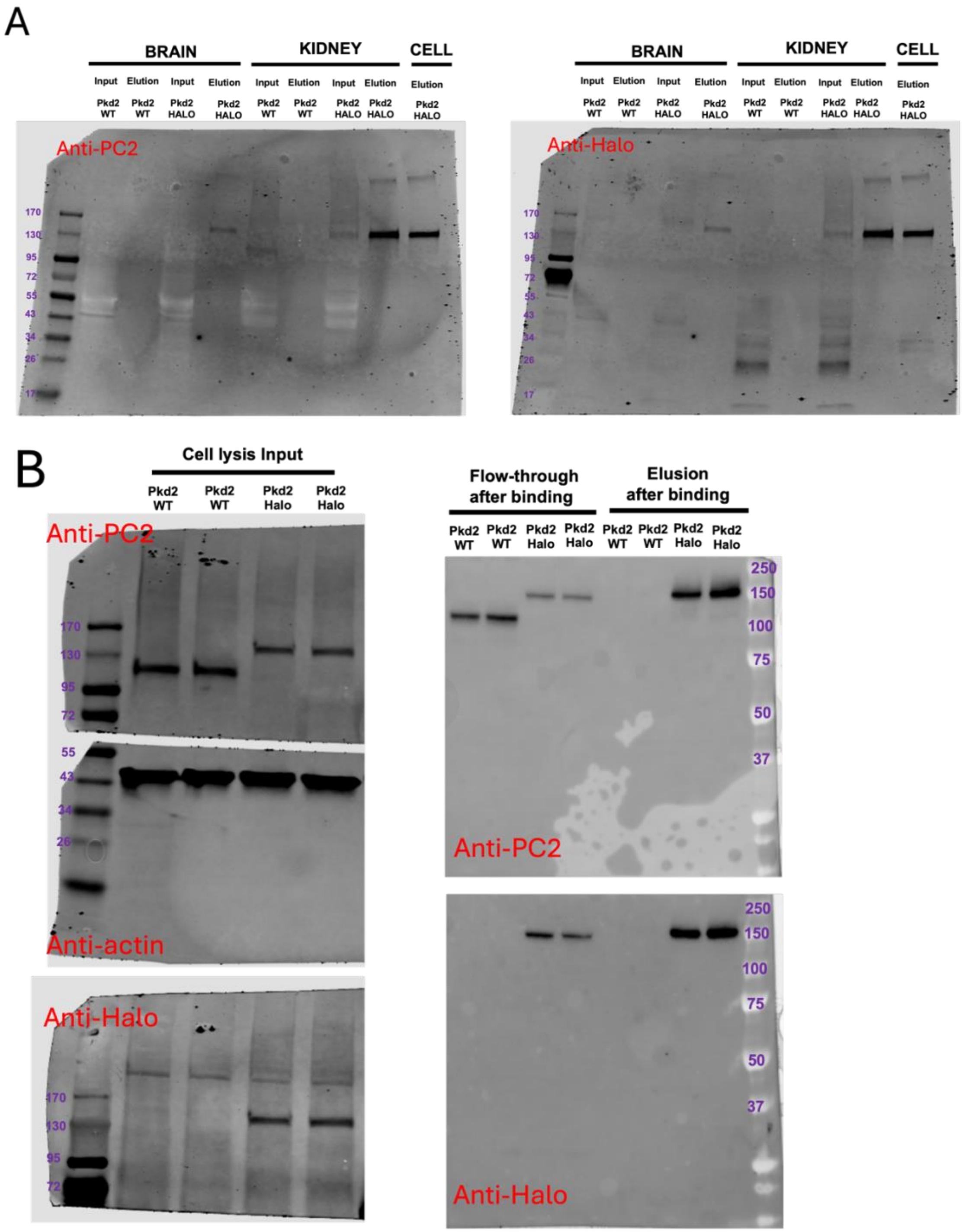

Supplemental Figure 4. Pkd2-c-Halo signal in choroid plexus from *Pkd2^c-Halo^* mice with varying doses of Halo-JFX554. While Halo-JFX554 (red) is visible in the choroid plexus cilia of *Pkd2^c-Halo^* mice, the signal is increased in samples injected with higher doses. The signal at 5 nmol/mouse and 10 nmol/mouse is similar suggesting saturation of the Pkd2-c-Halo protein at these doses. Nuclei are stained with Hoechst (blue) in top panels.

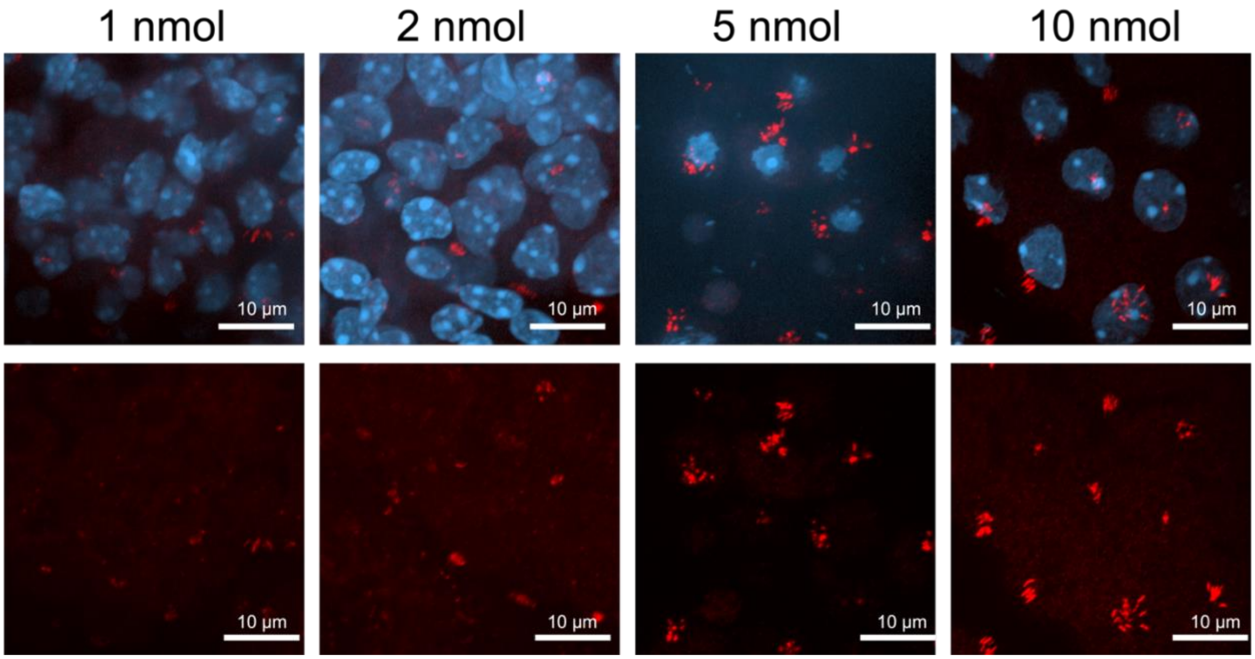

Supplemental Figure 5. (A) Representative confocal images of kidney sections stained with acetylated α-tubulin antibody (white) and PC2 (green) following injection of Halo-JF552 (red) used for quantification of co-localization of PC2 and Pkd2-c-Halo signal, white arrow indicates cilium. Adult-induced *CAGGCre*^{ER}*; Pkd2*$^{flox/flox}$ (12 weeks post tamoxifen induction, labelled as *Pkd2*^{KO}) was used as negative control for PC2 antibody validation. The arrows indicate primary cilia. Scale bar: 5 µm. **(B)** Representative confocal images of kidney sections stained with acetylated α-tubulin antibody (white) and Calnexin (green) following injection of Halo-JF552 (red) used for quantification of co-localization of ER and Pkd2-c-Halo signal. Scale bar: 5 µm.

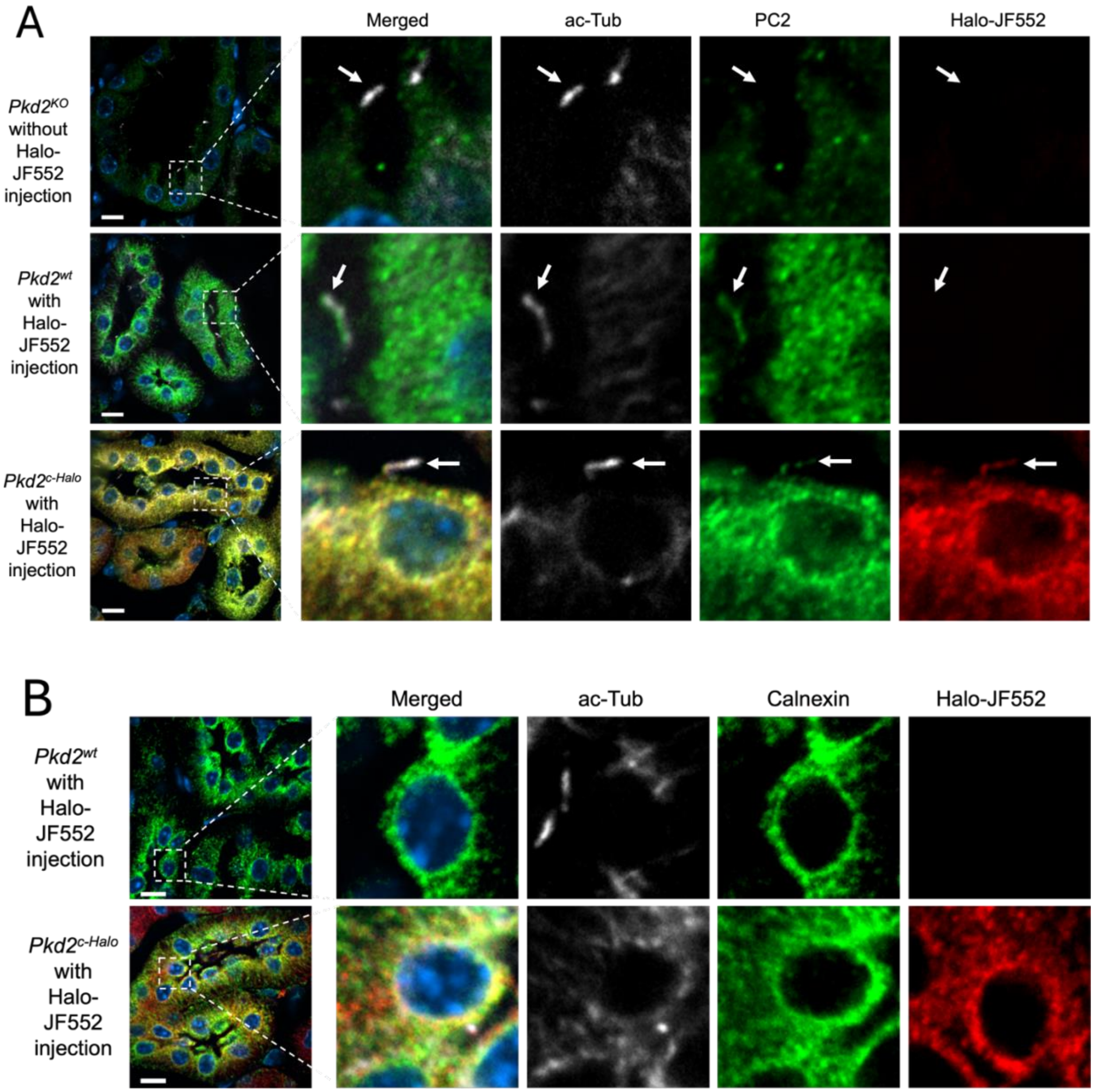

Supplemental Figure 6. Proximal tubule origin of *Pkd2*$^{c\text{-}Halo}$ cell line. Pkd2$^{\text{c-Halo}}$ cells were immunostained with markers for proximal tubule (Hnf4a), thick ascending loop of Henle (Uromodulin), and distal nephron segments (Pou3f3). The cells express the proximal tubule marker but are negative for proteins expressed in distal nephron segments.

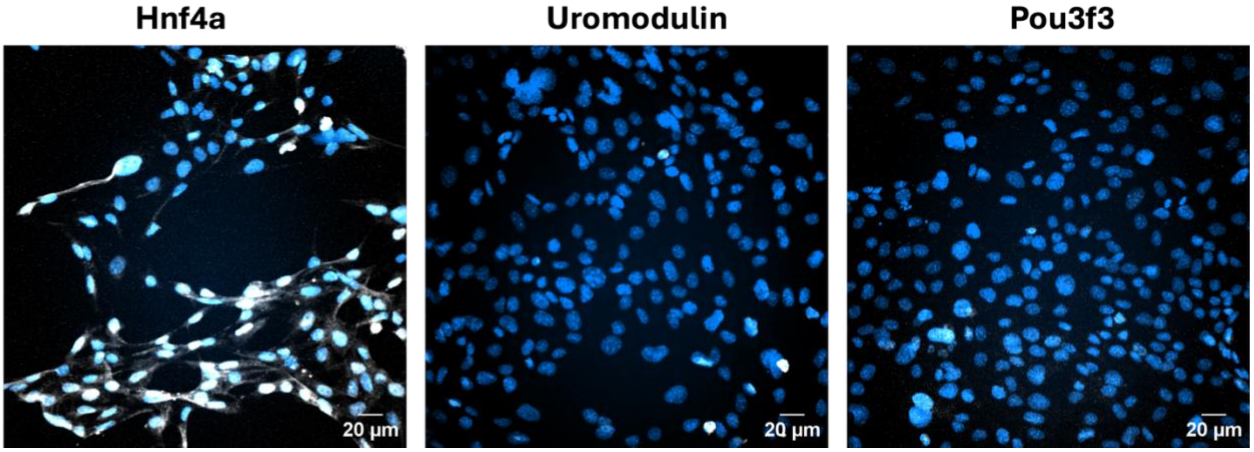

Supplemental Figure 7. Full length immunoblots of WB results for Figure 4A

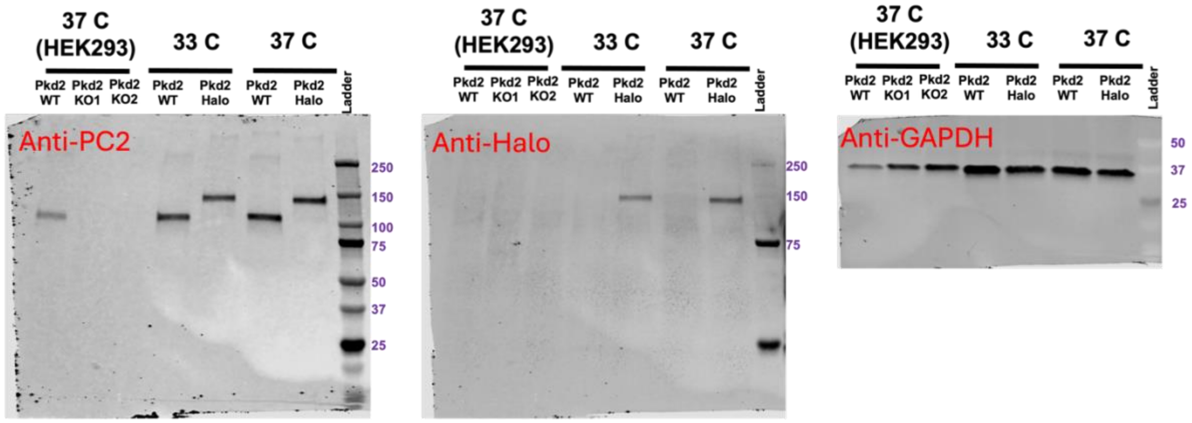

Supplemental Figure 8. Pkd2-c-Halo signal in *Pkd2$^{c\text{-}Halo}$* cell line with different concentrations of Halo ligands incubations. Halo-ligand is visible in the cytoplasm and cilia at concentrations as low as 12.5 nM although the signal intensity increases with concentrations up to 25 nM. No increase is observed beyond 25 nM suggesting this concentration achieves saturation of the available Pkd2-c-Halo protein.

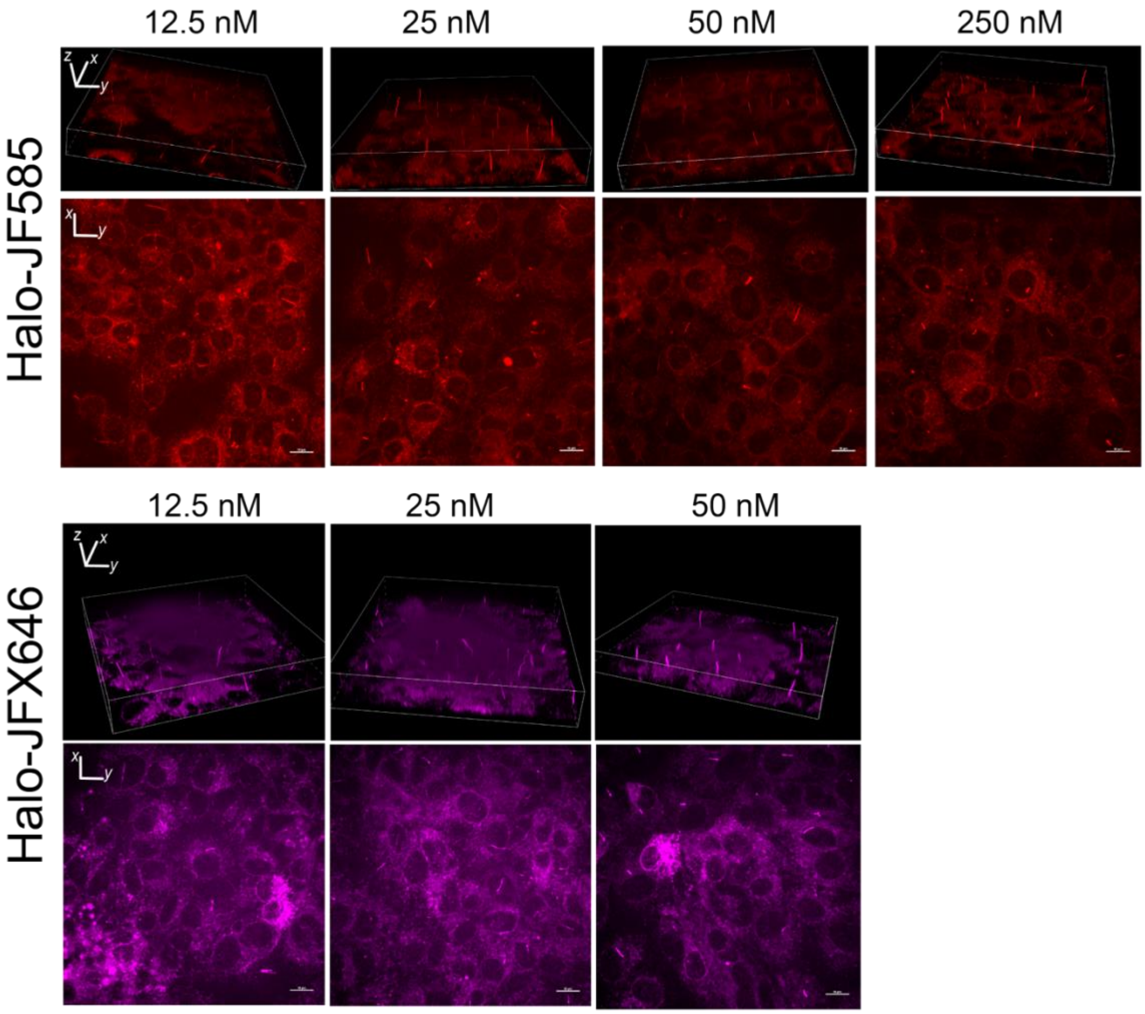

Supplemental Figure 9. Pkd2-c-Halo turnover in *Pkd2$^{c\text{-}Halo}$;Sstr3^{gfp}* cell line at 33°C. Cells were incubated with Halo-JF585 ligand to label all Pkd2-c-Halo, washed extensively then incubated with Halo-JFX646 and imaged live at the indicated times to visualize turnover of the Pkd2-c-Halo protein.

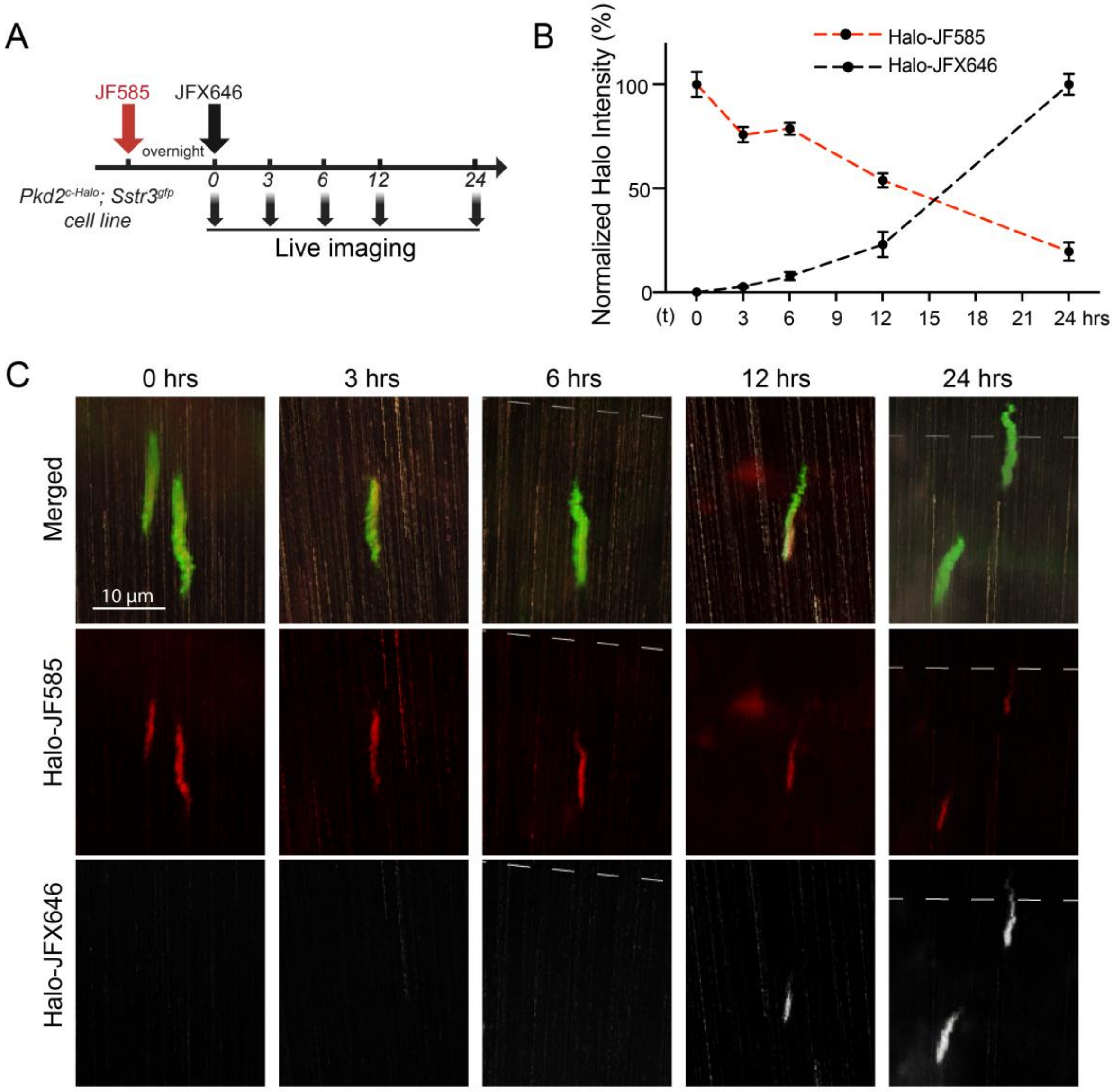

Supplemental Figure 10. Loss of ciliary localization of Pkd2-c-Halo in $Pkd2^{c\text{-}Halo}$;$Sstr3^{gfp}$ cells with Pkd1 deletion or Tulp3 mutation with live cell imaging

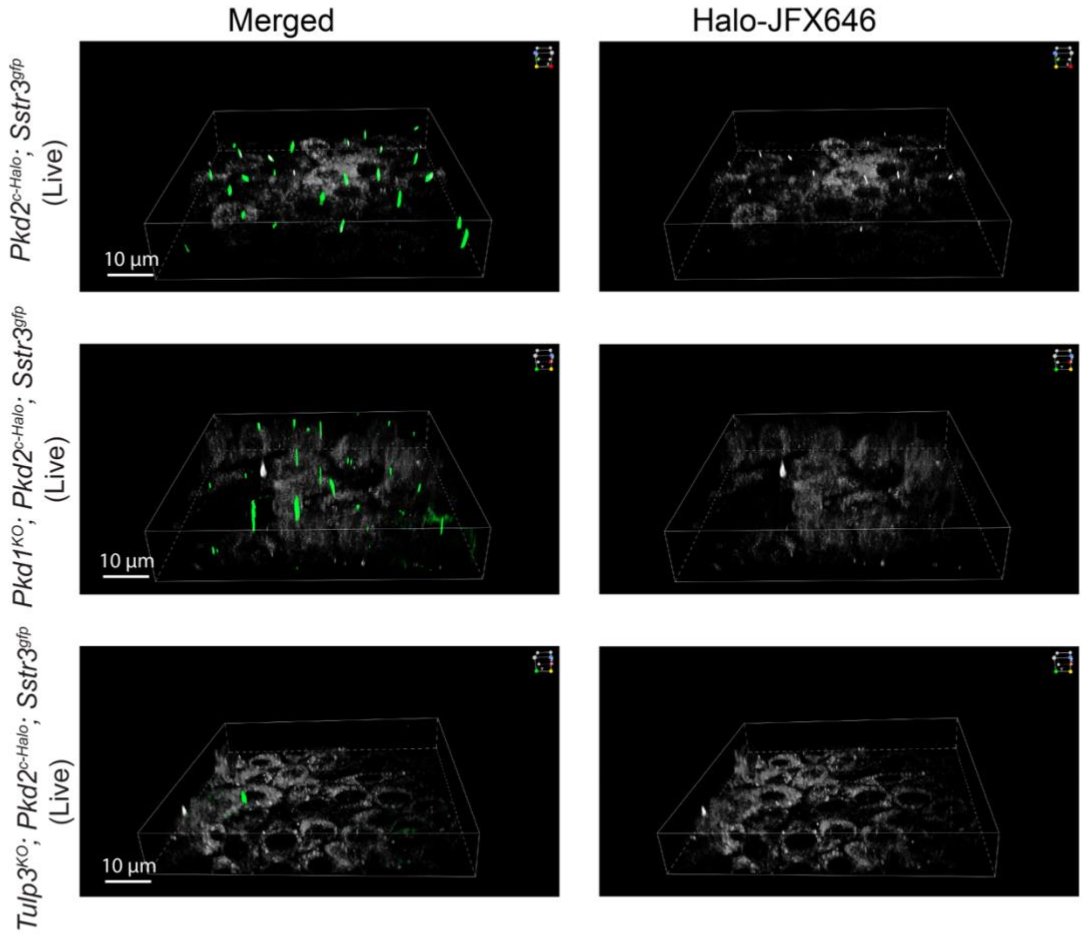

Video lists

Video 1. Pkd2-Halo kidney labeled with HaloTag ligand JFX554; Arl13b marks primary cilia. Related to Figure 3.

Video 2. Pkd2-Halo kidney labeled with HaloTag ligand JF552; LTL marks proximal tubules; acetylated α-tubulin marks primary cilia. Related to Figure 3.

Video 3. Pkd2-WT kidney incubated with HaloTag ligand JF552 (negative control); LTL marks proximal tubules; acetylated α-tubulin marks primary cilia. Related to Figure 3.

Video 4. Pkd2-WT; SSTR3-GFP cells incubated with HaloTag ligand JFX646. Related to Figure 4.

Video 5. Pkd2-Halo; SSTR3-GFP cells incubated with HaloTag ligand JFX646. Related to Figure 4.

Video 6. Non-injured Pkd2-Halo kidney labeled with HaloTag ligand JFX554; LTL marks proximal tubules; acetylated α-tubulin marks primary cilia. Related to Figure 5.

Video 7. UUO-injured Pkd2-Halo kidney labeled with HaloTag ligand JFX554; LTL marks proximal tubules; acetylated α-tubulin marks primary cilia. Related to Figure 5.

Video 8. IRI-injured Pkd2-Halo kidney labeled with HaloTag ligand JFX554; LTL marks proximal tubules; acetylated α-tubulin marks primary cilia. Related to Figure 5.

Video 9. Tulp3 mutant Pkd2-Halo kidney labeled with HaloTag ligand JFX554; LTL marks proximal tubules; acetylated α-tubulin marks primary cilia. Related to Figure 6.
